# Outcomes for an arboreal folivore after rehabilitation and implications for management

**DOI:** 10.1038/s41598-023-33535-y

**Published:** 2023-04-21

**Authors:** Kellie A. Leigh, Lacey N. Hofweber, Brienna K. Sloggett, Victoria L. Inman, Lachlan J. Pettit, Aditi Sriram, Ron Haering

**Affiliations:** 1 Science for Wildlife Ltd, PO Box 5, Mount Victoria, NSW 2786 Australia; 2 Department of Planning and Environment, National Parks and Wildlife Service, Locked Bag 5022, Parramatta, NSW 2124 Australia

**Keywords:** Ecology, Behavioural ecology, Conservation biology, Behavioural ecology, Conservation biology

## Abstract

Wildlife rehabilitation is a critical part of animal welfare that contributes to species conservation. Despite the resources that go into rehabilitation, how animals fare after release from care is unknown. This is particularly true for cryptic arboreal species where specialist diets in care and low detectability in the wild present challenges for both care and post-release monitoring. We evaluated post-release outcomes for koalas and assessed if koalas were fed appropriately while in care. We monitored 36 koalas that had experienced one of three categories of medical intervention (none, minor, major) during rehabilitation. We examined the drivers of (i) koala survival and (ii) movements post-release, and (iii) evaluated variation between the species of browse fed in care versus browse selected by koalas in-situ. Overall, the post release survival rate of koalas was 58.5%, with only koalas that received medical intervention experiencing mortality. A critical threshold for mortality occurred at two weeks post-release and mortality was related to the measurable indicators of low body condition and poor climbing ability at time of release. In the month following their release, animals translocated furthest from their capture point moved the furthest. There was poor overlap between the tree species that koalas were fed in care and those they utilized post-release. We provide recommendations to address critical gaps in rehabilitation practices, as well as priorities for monitoring animals post-release to improve outcomes for arboreal folivores.

## Introduction

Wildlife rehabilitation is the capture, treatment or care, and subsequent release of injured, sick, or orphaned wildlife^1^. Although rehabilitation often occurs at the scale of an individual animal, it can be an effective mechanism for species conservation^2,3^. Despite this, wildlife rehabilitation as a tool for conservation is rarely documented and can be underutilised by conservation scientists given it is often a volunteer-based sector^4^.

Wildlife rehabilitation is practiced globally^4^, at oftentimes large scales^5–7^. Given the extent and number of wildlife rehabilitation centres and programs worldwide, rehabilitation efforts comprise a significant amount of time and resources. In New South Wales (NSW), Australia, on average 78,259–104,000 animals are rescued annually with approximately 35% successfully rehabilitated, and this number is increasing over time^8,9^. Wildlife rehabilitation in NSW is conducted by over 6000 rehabilitators^8,10^, around a third of Australia’s total rehabilitators^11^. Rehabilitators volunteer, on average, 17 h per week caring for wildlife^8^ increasing to an average of 32 h per week when rehabilitating mammals, and in some cases up to 100 h per week^12^. Further, wildlife rehabilitators often personally finance this work, averaging between AUD$3000 and $5300 annually; up to AUD $24,000 on average over a rehabilitator’s career^12,13^. Accounting for their unpaid time and resources, it is estimated that wildlife rehabilitators in NSW alone contribute a minimum of AUD$27 million per annum^8^.

The time and financial cost of rescuing and rehabilitating wildlife varies based on the species and local protocols and policies. Rehabilitation efforts for koalas are particularly financially and physically intensive^14^ due to their specialist diet (koalas require access to fresh leaves from at least two eucalypt species at all times^15^), and sensitive gut microbiome and digestive physiology^16^. Koala rehabilitators regularly spend 15–20 h a week caring for koalas^10^ (J. Stark, personal communication, 2021) increasing to nearly 50 h per week if the koala is critically ill (E. Meadows, personal communication, 2021). Further, veterinarians often provide their assistance to assess and treat koalas and other wildlife species pro bono^17^.

Although significant resources are invested in wildlife rehabilitation, there is limited research on the rates, and drivers, of success of rehabilitated animals after they are released back into the wild^6,18–22^. Definitions of success after rehabilitation can vary, and understandably often consider survival rates of released animals, though generally only over relatively short time periods. However, beyond survival, an important consideration for many species is their post-release movements and if individuals re-establish home ranges or territories. In developed areas, where wildlife is exposed to anthropogenic threats, animals that move greater distances may have increased risks of road accidents and attacks from domestic animals^23,24^. Further, increased movements may be an indication that the animal is experiencing strong intra-specific competition^25^ (that has associated stress), or for some species a lack of conspecifics^26^, or that the animal has limited access to high quality or preferred habitats at its release site thereby increasing its energetic requirements to find food^27,28^. Increased movement after captivity or translocation is also common as a stress response^29–31^. At the population level, failure to establish a home range or territory could impact breeding rates^32,33^. An additional key factor for dietary specialists is their diet while in care and associated impacts on an animal’s post-release success.

By providing data to evaluate the success of rehabilitation practices, post-release monitoring should play a key role in examining and improving current rehabilitation practices^34^. However, the current literature has limited application in this regard, with most post-release monitoring studies focused on catastrophic events^35^, translocations^6,36,37^, or captive-reared orphaned animals^38–40^, and rarely include admission related to direct anthropogenic causes such domestic dog attacks and vehicle strikes. Relatively few post-release monitoring studies are conducted on mammals, particularly arboreal mammals, and the studies that exist generally examine a single indicator of post-release success; other than survival, these include release site^41,42^ or habitat, home range or movements^35^, reproduction^43^, or susceptibility to disease^44^. We found few studies that evaluated success using more than one outcome, that considered the medical treatment that the animal was given, or that compared ex situ diet to in situ diets^6,22^, all of which are highly relevant to informing management practices.

The paucity of rigorous post-release monitoring studies has been largely attributed to insufficient funding^4^, with the wildlife rehabilitation sector relying heavily on volunteers^8,9^. Successful long-term monitoring requires sometimes costly equipment, large time investments, and a high level of survey or skill^45^. Difficulty of monitoring varies by species^46,47^, with arboreal mammals particularly difficult given they are often cryptic in nature, often nocturnal, and are difficult to detect using traditional survey methods^45^.

Koalas (*Phascolarctos cinereus*) are one such cryptic arboreal mammal species, for which there is limited information on post-rehabilitation success. Koalas are an iconic Australian animal; acting as a flagship species that generates public interest in conservation and as an umbrella species^48^, with conservation of their forest habitats benefitting multiple species within the broader ecosystem^49^. Koalas are listed as Endangered within NSW, Queensland (QLD) and the Australian Capital Territory^50^. Approximately two-thirds of the species’ range is contained in NSW and QLD, and while empirical data are lacking for many populations, by 2016 koala numbers in each state were estimated to have declined by 26% and 53% respectively^51^. The key factors contributing to koala population declines are habitat loss and fragmentation, which then drive other threats such as vehicle strike, dog attack and increased susceptibility to disease, including chlamydial infection^21,43,52–55^. Climate change is driving more extreme weather events, such as the 2019–2020 bushfires across the eastern states of Australia^56^ which led to further declines in koala populations^57^. It is estimated that 11% of suitable koala habitat was impacted during the fires^58^. Koala admittance to wildlife hospitals had already been increasing over time^9,52^, and following the bushfires increased further^10,21^. Despite theoretical evidence that rehabilitated animals may contribute to population growth^3^, there is limited empirical data on post-release outcomes for rehabilitated koalas or similar species (e.g., brushtail possums^59^) to guide investment and decision making in population management.

To address these critical information gaps and evaluate the effectiveness of current koala rehabilitation protocols, we examined the drivers of (i) koala survival and (ii) movements post-release, and (iii) evaluated variation between the species of browse fed in care versus browse selected by koalas in situ.

## Materials and methods

### Study area

**Figure 1:**
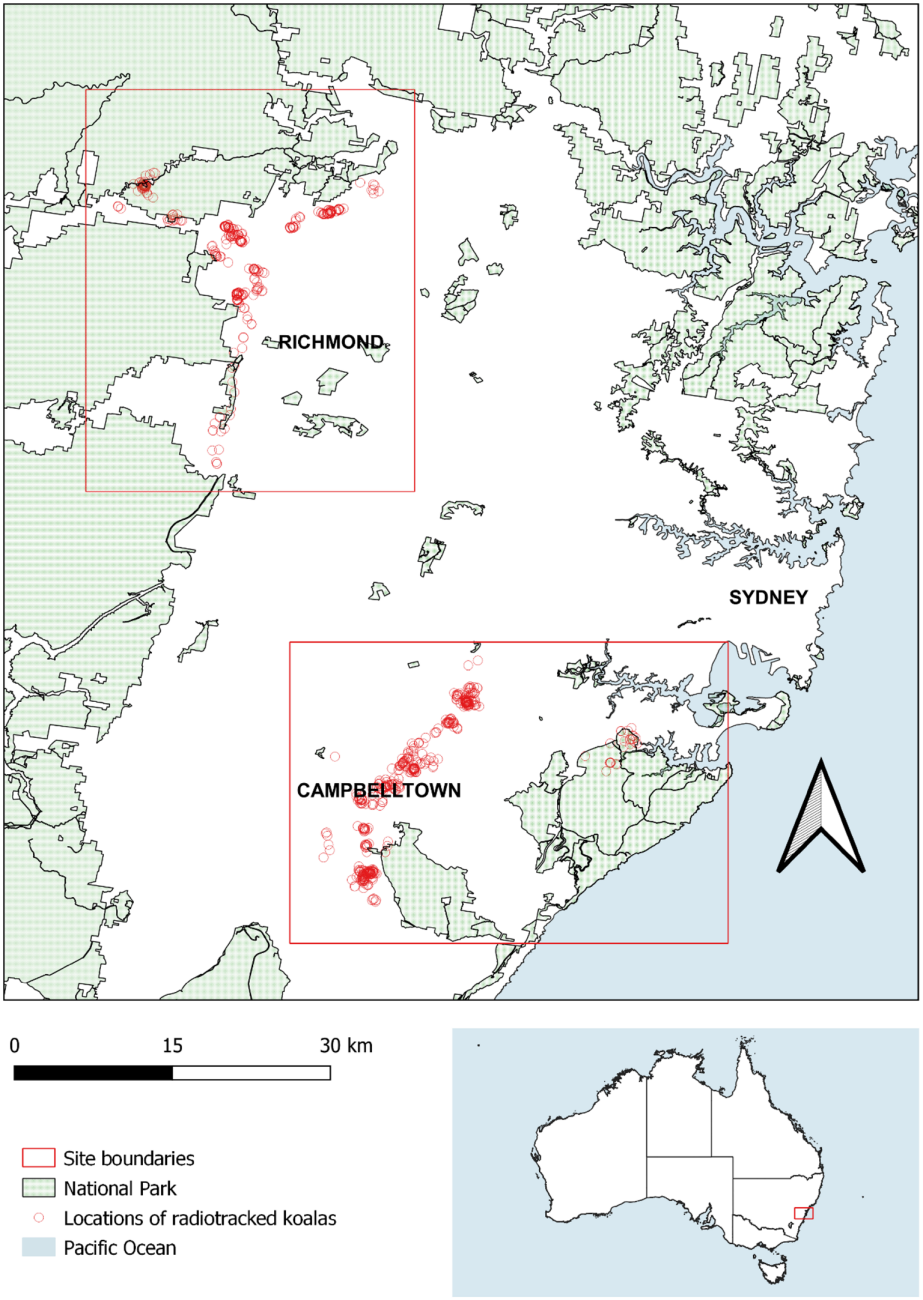
The Northwest and Southwest study sites within the Greater Sydney Region, NSW, Australia.

The study was conducted between 1 December 2019 and 30 December 2021 in the Greater Sydney Region of New South Wales, Australia, an area heavily impacted by human development. The study area was divided into two sites (Northwest and Southwest) (Fig. 1).

### Koala capture and rehabilitation

The NSW Department of Planning and Environment (DPE) provides the Code of Practice for Injured, Sick and Orphaned Koalas (the Code of Practice), which contains both standards and guidelines for the care of koalas^15^. Koalas that were admitted to one of five treatment facilities for initial consultations and then placed with four volunteer wildlife rehabilitators for further care and treatment were included in the study. We obtained records from wildlife rehabilitators and veterinarians on the location of capture, reason for admission, veterinary diagnosis, veterinary treatment, age (< 3 years, subadult; > 3 years, adult) based on tooth wear class^60^, time in care, rehabilitation measures, and (if available) feed tree species supplied to koalas during care.

### Assigning medical intervention categories

These koalas were categorised either as ‘minor’, if they received minor medical intervention (e.g., water for dehydration, topical creams), or ‘major’, if they received major medical intervention (e.g., surgery, intravenous fluids for dehydration, oral antibiotics, *Chlamydia* treatment). The Code of Practice recommends that all captured koalas are screened for *Chlamydia* prior to release, and so all koalas that were admitted to a treatment facility (i.e., ‘minor’ and ‘major’ koalas) were tested for *Chlamydia*, which requires sedation. In addition, we included in the study koalas that were captured due to risk of injury (e.g., along a busy road, in an unsafe tree, or in close proximity to a domestic dog), but were not admitted to a treatment facility (and therefore were not screened for *Chlamydia*) and did not receive medical intervention (categorised as ‘none’), though may have still spent time in care for monitoring. Some koalas had a previous history of care.

### Koala release, radio-tracking, and recapture

Koalas were deemed fit for release back into the wild by veterinarians or experienced koala rehabilitators^15^. Prior to release, koalas were fitted with either one or two VHF ear tags (modified 11 g bird/bat transmitter model R1-RCM, Holohill Canada) or a VHF collar (custom design, 80 g, Lotek New Zealand). Koalas were then independently assessed at the time of release by the same researcher, weighed, aged using a tooth wear class^60^, and given a body condition score using a standardised scale^61^ from one (emaciated) to five (excellent), but modified to include 0.5 intervals. The Code of Practice states koalas should only be released if their body score is three or higher, however our independent assessment noted four koalas released with body condition scores lower than this threshold. At the time of release, the same observer assessed climbing ability scored as one (very poor—could not climb tree unassisted), two (moderate—slow to climb tree), or three (excellent—easily climbed tree), including 0.5 intervals, and observed until the koala had climbed and settled in the tree.

**Figure 2:**
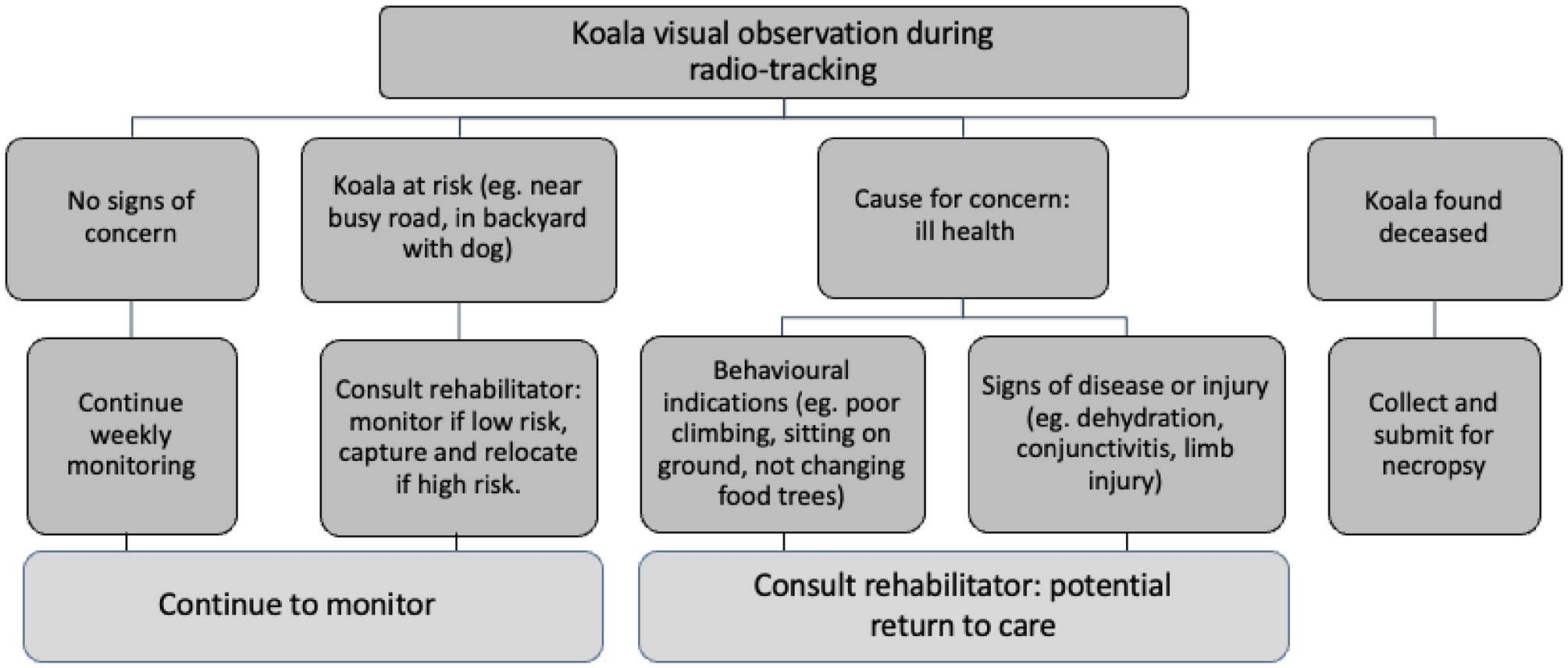
The decision-making process if any causes of concern or risks to koalas were encountered during weekly monitoring and visual inspection by radio tracking.

Released koalas were radio-tracked approximately once per week and their location recorded using a handheld GPS (GPSMAP 64 s, Garmin, USA), along with the tree species the koala occupied. If a koala was in an area which couldn’t be accessed (e.g., private property) a vicinity waypoint was recorded as close to the koala as possible. Since this was an observational study, during radio-tracking if koalas showed signs of poor health a wildlife rehabilitator was called to assess the koala to make the decision on whether the koala should be recaptured and re-admitted to a treatment facility in accordance with the Code of Practice. The decision making process for different scenarios is outlined in Fig. 2. Poor health was assessed using any of the following criteria: poor climbing ability, failure to move between food trees, koala remaining on the ground, displaying abnormal behaviour or signs of stress such as panting, evidence of dehydration or condition loss, excessive head lifting or drinking water, eye discharge, or obvious injuries or illness including signs of *Chlamydiosis* such as wet bottom and/or conjunctivitis^15,62^. The full history of each koala is provided in Table S7. Koalas were sometimes recaptured using the flag and restraint rope method^1,2^ to perform health assessments, record body condition, and check for external signs of disease or injury but were released within one hour at the point of capture with no time spent in care.

### Ethics declaration

All procedures involving animals were approved by a NSW registered Animal Ethics Committee (AEC); the University of Sydney Animal Ethics Committee to June 2021, then NSW Department of Primary Industries Secretary’s Animal Care and Ethics Committee until the end of the project. All activities were carried out under a Scientific Licence (Number SL101364) from the NSW National Parks and Wildlife Service, Department of Planning and Environment, and all methods were carried out in accordance with the guidelines and regulations set by these AECs and the scientific licencing authority^63^, which are in accordance with ARRIVE guidelines.

### Analysis

Throughout, averages are presented ± standard error and statistics were conducted in R (R Core Team, 2021) unless otherwise stated.

### Differences between capture and release locations

The Code of Practice specifies that if the area is suitable koalas should be released back to where they were captured, otherwise released as close as possible to the capture site in a suitable environment. Accordingly, we examined differences between koala capture and release locations. We measured the distance between koala capture and release sites as the straight-line distance between the two sites using the pointDistance function in the raster package^64^. We considered differences in soil types, koala habitat suitability, and vegetation. Soil types were taken from the Great Soil Group (GSG) Soil Type map of NSW^65^, habitat rankings from the Koala Habitat Suitability Model (KHSM)^66^, which ranks vegetation between 0 (poor quality) to 1 (good quality), and vegetation type from one of five vegetation maps (see Text S1 for explanation of how vegetation maps were ranked). These variables were extracted from the capture and release sites (buffered 10 m to account for GPS inaccuracy) using the st_intersection function from the sf package^67^ for soil and vegetation, and the exact _extract function from the exactextractr package^68^ for KHSM, calculating the average value (for KHSM) and the value with the largest area (for soil and vegetation) within the buffer zone. Similar vegetation types were grouped where possible into vegetation groups based on the presence of koala food trees, where there was 100% concordance for primary food trees, combined with > 75% match between secondary food trees (Table S1). Areas mapped as cleared, modified, plantation, weed, non-native, or planted were categorised as ‘modified vegetation.’

### Post-release movements

To assess koala movements immediately after release, we examined the daily distance that koalas moved from their release site in the first 30 days using radio tracking locations. The distance between subsequent locations was measured as the straight-line distance between the two points (measured as above) and divided by the number of days between locations to get an estimate of daily distance moved. We examined what factors were the strongest drivers of koala daily distance moved within the first 30 days by fitting a generalised linear mixed model, family Gamma (with log link) with koala ID as a random variable, and the following explanatory variables: days since release (second-order polynomial, as we predicted movement would change over time in a potentially non-linear way), distance between captures and release sites (logged), days in care (logged), age, sex, medical intervention, differences in soil types, koala habitat suitability, and vegetation. We also considered first and second order interactions of these variables with days since release (see Text S2 for full model). We used the ‘dredge’ function in the ‘MuMIn’ package^69^ to automatically generate models with all possible combinations of the explanatory variables. We then used an information-theoretic approach to identify the best-fitting models by considering models within ΔAICc ≤ 2. If multiple models had ΔAICc ≤ 2, we used a model averaging approach using the ‘model.avg’ ('MuMIn' package^69^) and ‘summary’ functions. For all top selected models, we confirmed the assumptions of homogeneity of variance and normality of data were met using the ‘simulateResiduals’ function (‘DHARMa’ package^70^, examining plots of distributions of residuals against the predictors and Q–Q plots of the normal distribution.

Further, we examined the maximum distance that koalas occurred from their release site within the first 30 and the first 100 days following release. For this, we only considered koalas that had at least 30 days and 100 days tracking data, respectively. We fit a linear model with the maximum distance from release site (logged) as the response variable and the following explanatory variables: distance between captures and release sites (logged), days in care (logged), age, sex, medical intervention, differences in soil types, koala habitat suitability, and vegetation. Given the limited data, we did not consider interactions. Model selection and interpretation was conducted as above.

For both variables (daily distance moved and maximum distance from release site), we only included vicinity points in calculations if they had an estimated accuracy of 100 m or less, and calculations were only conducted for the first-time koalas were released (not subsequent releases).

### Post-release survival

Koala mortality was recorded at the time of the event if in situ, otherwise if a koala was recaptured and either died in care or required life-saving (i.e., major) medical intervention, a mortality event was recorded at the date of recapture. We used the dates of recaptures since mortality in the wild would be assumed to occur more rapidly (though we note that the stress of capture and captivity may influence mortality outcomes^71^). Koalas that survived or where tracking ceased early due to collar removal, loss of VHF transmitter, or lost signal were placed in the same category as koalas that survived. For this category, days to survival was based on the last day that koalas were tracked, minus the total number of days in care. We used data regardless of the number of capture and release events and assigned the medical intervention category for each koala for the survival analysis based on the highest level of medical intervention it had received during the study. For example, if a koala was originally captured and received no medical intervention, but subsequently was recaptured and received minor medical intervention, for the survival analysis it was considered a ‘minor’ koala.

To estimate the probability of koala survival post-release, we used the Kaplan Meier Survival estimate^72^ in GraphPad Prism V9.4 (GraphPad Software, San Diego, California USA). Post-release survivorship was calculated from the time of the koala’s first release. Survival curves were assessed over two time periods: acute survival (within 30 days after release) and long-term survival (entire duration of their time in the study). Comparisons were made between the probability of survival with respect to medical intervention (none, minor, major), age (subadult versus adult), region (Northwest versus Southwest), and year [the year the koala was released: Year 1 (2020) and Year 2 (2021)]. Differences in survival between medical intervention categories were tested for significance using Gehan-Breslow-Wilcoxon with a Bonferroni correction applied to account for multiple comparisons. All other survival comparisons were tested for significance using the Mantel Cox-Log Rank Test^72^.

Further, we examined the relationship between survival (acute and long-term) and four continuous variables [climbing ability score, body condition score, distance between capture and release sites (logged), and days in care (logged)], by fitting generalised linear models, family binomial, with the glmmTMB function from the glmmTMB package^73^. Given the limited mortality data, fitting of complex models was not possible and therefore we fit univariate models for survival against each variable, which allowed greater statistical power. We compared models using AICc and determined the significance of the effects using the ‘summary’ function. As above, we confirmed model assumptions were met.

### Habitat use and diet suitability

The diurnal tree species selected by koalas following release (as determined from radio-tracking) were compared to the tree species the koalas were fed in care. We removed tree use data where direct human intervention may have impacted the koala tree selection (e.g., the tree the koala was released in), where the species was not identified, or where the species occupied was not from *Eucalyptus, Corymbia*, or *Angophora* genera, unless that species had been observed in koala diets (e.g., *Syncarpia glomulifera*) (unpublished data). We excluded koalas that were released the same or following day after their capture, since any impact on diet fed in care would be minimal. Koala diurnal tree use was used as a surrogate measure for diet preference, based on data from two different studies of koalas in the region; one showing that diurnal tree use and diet composition tested by DNA sequencing was comparable^74^ (all species found in the diet were used regularly diurnally), and one where koalas had been observed feeding from each of the tree species they occupied most frequently during the day based on tracking data (> 60% occurrence, unpublished data).

Data on the composition of tree species koalas were fed while in care was comprised from a list of the available feed tree species rehabilitators collected branches from at each browse collection site, along with the frequency that each site was visited throughout the year (from twice per week to once every eight weeks). Identification of the available feed tree species was ground-truthed by a researcher during site visits to each browse collection site. Data on the specific tree species fed to each koala were unavailable. Therefore, based on interviews of rehabilitators who cited consistent browse collection across all feed trees within their individual browse collection sites we assumed the same species of browse was collected and offered to koalas.

Since rehabilitators selected more than one tree species each day they collected browse, we calculated the relative frequency that each tree species was collected by each rehabilitator (and offered to koalas) by multiplying the number of sites each species was collected from by the frequency of collection, calculated over the maximum collection frequency period (eight weeks). For example, a tree species collected at three different sites all with weekly frequency would score a relative frequency of 24 over eight weeks. Koalas were recorded in only one tree species on any day, so we calculated the number of times a koala occupied each tree species and combined the data for all koalas under each rehabilitator. We conducted a linear regression in Microsoft Excel to assess the relationship between the number of koala locations and tree species richness for all koalas.

Tree species were categorised into three groups for analysis based on their importance to koalas, as either primary, secondary, or tertiary food trees (Table S2). Grouping was based on known koala food trees^75^ as well as site-specific data on koala tree use frequency and observed feeding behaviour^74,76^ [and unpublished data]. We used chi-square tests in GraphPad to compare the relative proportions of tree species used by koalas in situ with the relative proportion of tree species fed in care to those koalas, by each rehabilitator.

Due to the limitations around koala diurnal tree use data, and in case koalas fed from different tree species at night, we also looked at vegetation communities to assess the general availability of different tree species to koalas versus the species the rehabilitators used to collect browse from. We identified the vegetation communities that koala locations occurred in using the Point Sampling Tool in QGIS and combined the data for koalas by rehabilitator. We only used data from koalas where identification of the tree species used by the koala was confirmed, where koalas had 5 or more tracking locations after release to reduce the impact of any early displacement movements, and we did not include the initial release location. The tree species contained in each vegetation community were determined by reviewing the associated map reports (Table S1) and we included all *Eucalypt*, *Angophora* and *Corymbia* species that were listed as dominant or diagnostic tree species since these were the most likely species to occur consistently across the vegetation community. We omitted the vegetation classifications that included planted canopy trees from the data, since these areas were human modified and included tree species with no local provenance. For each rehabilitator we then evaluated the number of tree species available to the koalas in situ that were also used for browse collection, as well as the number that were used for browse collection but that were not available to the koalas in situ.

## Results

Based on records received from rehabilitators and veterinarians during the study, there was an overall mortality rate of approximately 53% for koalas captured and taken into care (n = 142). Our post-release study included 36 released koalas; six koalas that received no medical intervention, 16 koalas that received minor medical intervention, and 14 koalas that received major medical intervention. These categorisations represent the medical interventions for the first time the koalas were captured. Seventeen of the 36 koalas were captured more than once during the duration of the study, either to be brought back into care for further treatment or to be relocated; 12 koalas were captured twice, three koalas were captured three times, one koala was captured five times and another koala six times. Detailed histories for each koala are included in Supplementary Information (Table S7). There were more males (22) than females (14) included in the study, more adults (25) than subadults (11), and more koalas in the Southwest site (25) than the Northwest site (11) (Table S3). Considering the first time they were captured for the study, koalas that did not receive any medical intervention were initially kept in care for an average of 1.8 ± 0.9 days (range 0–6 days), minor koalas for 36.3 ± 22.9 days (range 2—378 days), and major koalas for 95.3 ± 12.5 days (range 9–154 days). Eight koalas did not receive any veterinary assessment upon their first capture during the study, though three were given oral fluids for dehydration (and therefore classed as minor intervention), with one of these koalas later dying.

### Differences between capture and release sites

The distance between capture and release sites varied, with an average distance of 1395 ± 300 m (range 23–9104 m). There was a difference in the vegetation group and soil type at the release site compared to the capture site for 86% and 31% of koalas, respectively. Based on the Koala Habitat Suitability Model, 39% of koalas were released onto less suitable habitat compared to their capture site.

### Post-release movements

**Figure 3:**
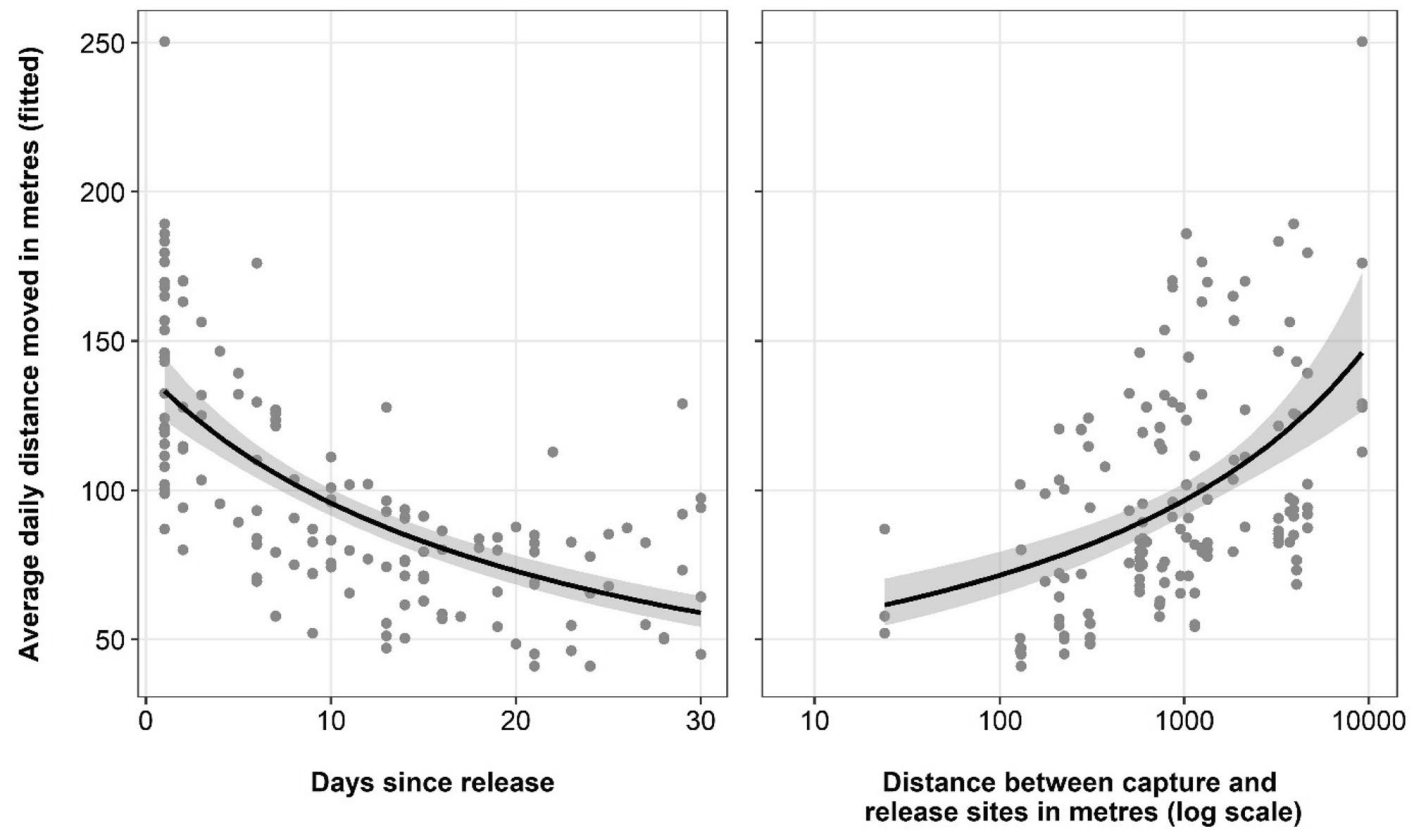
Relationships between days since release and distance between capture and release sites on fitted average daily distance moved by koalas from model-averaging of the top (∆AIC ≤ 2) generalised linear mixed models.

There were seven top models that best explained variation in daily distance moved by koalas in the 30 days following their release (Table S4). All models included the distance between the capture and release sites (logged) and days since release. Other variables included in the models were age, sex, difference in soil and vegetation between the capture and release sites, and the interaction between age and days since release. After model averaging, there was a significant positive relationship between daily movements and the distance between the capture and release sites (*p* = 0.019) and a negative linear relationship between days since release and movement (*p* = 0.006; Fig. 3, Table 1). The second order polynomial for days since release was not significant, nor were the other variables included in the model (all *p* < 0.05).

**Table 1:** Summary output from model averaging the top (∆AIC ≤ 2) generalised linear mixed models showing relationships (conditional average) with daily distance moved by koalas in the 30 days after release.

Explanatory variables	Estimate	Std. error	Adjusted std. error	z value	*p* value
Daily distance moved in first 30 days					
(Intercept)	3.096	0.586	0.591	5.239	< 0.001
Distance between capture and release (log)	0.190	0.080	0.081	2.353	0.019*
Poly (days since release)_1_	− 3.105	1.125	1.135	2.735	0.006*
Poly (days since release)_2_	1.376	1.397	1.406	0.978	0.328
Age_SUBADULT_	− 0.298	0.223	0.225	1.325	0.185
Sex_MALE_	0.218	0.213	0.214	1.015	0.310
Soil type of capture and release_SAME_	0.172	0.219	0.221	0.775	0.438
Age_SUBADULT_:poly (days since release)_1_	1.531	2.298	2.320	0.660	0.509
Age_SUBADULT_:poly (days since release)_2_	− 3.791	2.272	2.293	1.653	0.098
Vegetation type of capture and release_SAME_	0.209	0.358	0.361	0.578	0.563

Significant relationships are identified by asterisks. The categorical reference groups are: adult for age, female for sex, different for soil type of capture and release, and different for vegetation type of capture and release.

There were three models that best described the maximum distance that koalas moved in the first 30 days following release (Table S5). After model averaging, there was a positive relationship between the distance between capture and release sites (logged) and the maximum distance koalas were from their release site (*p* = 0.036; Table 2). Considering the maximum distance that koalas moved from their release site in the first 100 days, there were five models with ΔAICc ≤ 2, but these included the null model (Table S6), indicating there was little evidence of any variables being strong predictors of distance moved from release site.

**Table 2:** Summary output from model averaging the top (∆AIC ≤ 2) generalised linear mixed models showing relationships (conditional average) with the maximum distance koalas moved from their release site within 30 days after release from rehabilitation.

Explanatory variables	Estimate	Std. error	Adjusted std. error	z value	*p* value
Maximum distance from release site in first 30 days					
(Intercept)	4.494	0.981	1.033	4.350	< 0.001
Distance between capture and release (log)	0.335	0.152	0.160	2.096	0.036*
Medical intervention_MINOR_	− 0.535	0.405	0.430	1.244	0.213
Medical intervention_NONE_	0.680	0.564	0.598	1.136	0.256
Sex_MALE_	0.365	0.365	0.386	0.946	0.344

Significant relationships are identified by asterisks. The categorical reference groups are: major for medical intervention, and female for sex.

### Survival probability

Nine koalas were recorded as a mortality during the study: six koalas died and three returned to care and received a life-saving medical intervention (one of these subsequently died). Eight of the mortality events occurred in male koalas, eight were adult koalas, and five occurred within the first 11 days after release (see Table S7 for mortality details).

### Acute survival

**Figure 4:**
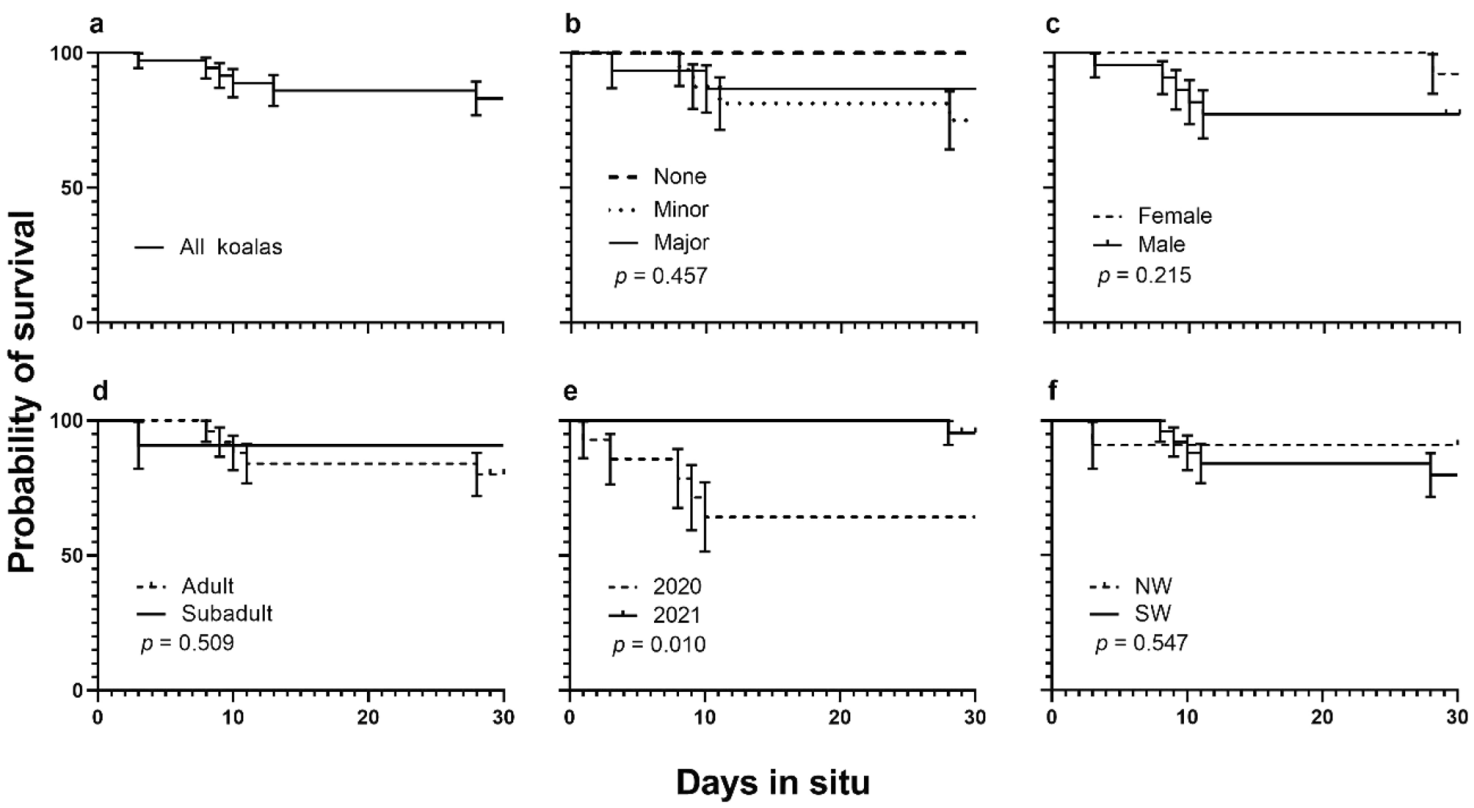
Kaplan Meier survival probability curves for acute survival for (**a**) all koalas, (**b**) considering medical intervention (none, minor, major), (**c**) sex (female, male), (**d**) age (adult, subadult), (**e**) study year (2020, 2021), and (**f**) region (northwest [NW], southwest [SW]).

Six of the 36 koalas experienced a mortality event within 30 days of being released from care indicating a survival rate of 83.2% ± 6.2 (Fig. 4a). Medical intervention was not significant (*X*^2^_2_ = 1.566, *p* = 0.457; Fig. 4b), nor was survival between male and female koalas (*X*^2^_1_ = 1.537, *p* = 0.215; Fig. 4c), or age (*X*^2^_1_ = 0.509, *p* = 0.476; Fig. 4d). Koalas in 2020 had a significantly lower probability of surviving than koalas in 2021 (64.3% ± 12.8 vs 95.5% ± 4.4; *X*^2^_1_ = 6.641, *p* = 0.010; Fig. 4e). The survival curves did not significantly differ based on region (*X*^2^_1_ = 0.547, *p* = 0.460; Fig. 4f).

Examining acute survival against the continuous variables, the strongest models were climbing ability (AICc = 30.169) and body condition scores (AICc = 30.385), with koalas with stronger climbing abilities and better body condition more likely to survive (*p* = 0.017 and *p* = 0.037, respectively; Table 3). The distance between capture and release sites and days in care were not significant predictors of acute koala survival (*p *= 0.430 and *p* = 0.611, respectively; Table 3).

**Table 3:** Summary output for models examining climbing ability, body score, distance between capture and release sites, and days in care against acute survival.

Model AICc	Explanatory variables	Estimate	Std. error	z value	*p* value
Acute koala survival					
30.169	(Intercept)	− 2.076	1.505	− 1.380	0.168
	Climbing ability	1.605	0.672	2.387	0.017*
30.385	(Intercept)	− 6.762	3.842	− 1.760	0.078
	Body condition	2.639	1.265	2.087	0.037*
36.547	(Intercept)	0.432	2.328	0.186	0.853
	Distance between capture and release (log)	0.182	0.357	0.508	0.611
36.166	(Intercept)	0.959	0.893	1.073	0.283
	Days in care (log)	0.231	0.292	0.790	0.430

Significant relationships are identified by asterisks.

### Long-term survival

**Figure 5:**
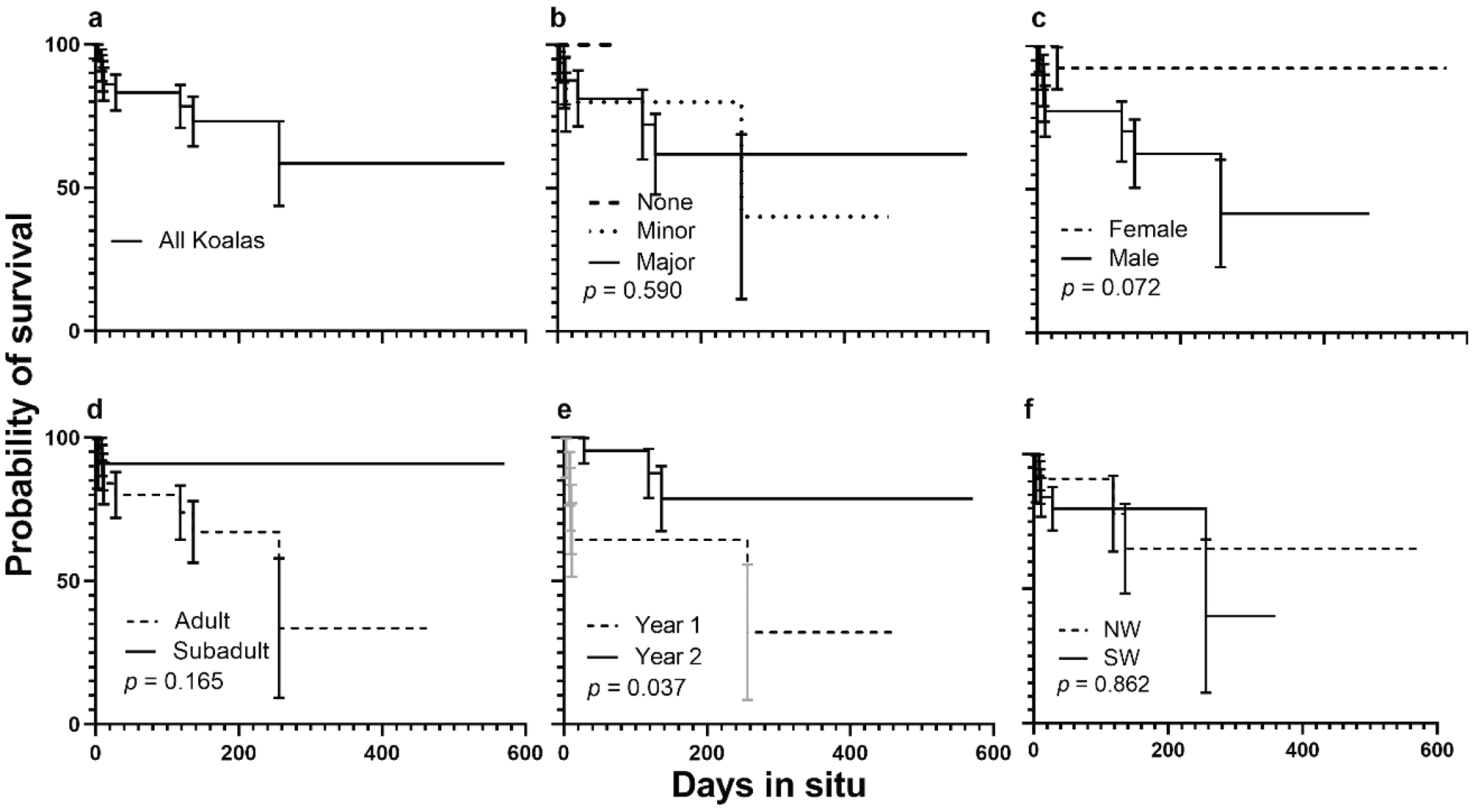
Kaplan Meier survival probability curves for long-term survival for (**a**) all koalas, (**b**) considering medical intervention (none, major, minor), (**c**) sex (female, male), (**d**) age (adult, subadult), (**e**) study year (year 1, year 2), and (**f**) region (northwest [NW], southwest [SW]).

The overall long-term survival for koalas was 58.5% ± 14.8 with eight confirmed mortalities (Fig. 5a). There was no significant difference in survival between koalas experiencing various levels of medical intervention (*X*^2^_2_ = 1.055, *p* = 0.590; Fig. 5b), nor a difference in survival between females (92.3% ± 7.4) and males (41.6% ± 18.8; *X*^2^_1_ = 3.242, *p* = 0.072; Fig. 5c). Koalas that were released in 2020 had significantly lower survival (32.1% ± 23.6) compared to those released in 2021 (78.8% ± 11.4; *X*^2^_1_ = 4.338, *p* = 0.037; Fig. 5e). There was no difference in survival depending on age (*X*^2^_1_ = 1.931, *p* = 0.165; Fig. 5d) and region (*X*^2^_1_ = 0.030, *p* = 0.862; Fig. 5f).

Examining long-term survival against the continuous variables, the strongest model included climbing ability score (AICc = 34.707), with koalas displaying stronger climbing abilities more likely to survive long-term (*p* = 0.006; Table 4). Further, koalas with better body condition were more likely to survive (*p* = 0.046; Table 4), and some evidence koalas released further from their capture point were more likely to survive longterm (*p* = 0.063; Table 4). There was no significant relationship between days in care and survival (*p* = 0.938; Table 4).

**Table 4:** Summary output for models examining climbing ability, body score, distance between capture and release sites, and days in care against long-term survival.

Model AICc	Explanatory variables	Estimate	Std. error	z value	*p* value
Long-term koala survival					
34.707	(Intercept)	− 3.390	1.653	− 2.051	0.040
	Climbing ability	1.886	0.689	2.739	0.006**
39.858	(Intercept)	− 4.826	2.909	− 1.659	0.097
	Body condition	1.814	0.911	1.991	0.046
40.769	(Intercept)	− 3.155	2.266	− 1.392	0.164
	Distance between capture and release (log)	0.669	0.360	1.858	0.063
44.846	(Intercept)	1.041	0.830	1.254	0.210
	Days in care (log)	0.019	0.247	0.078	0.938

Significant relationships are identified by asterisks, near significant (0.075 > p > 0.05) relationships identified by dots.

### Diet suitability

**Figure 6:**
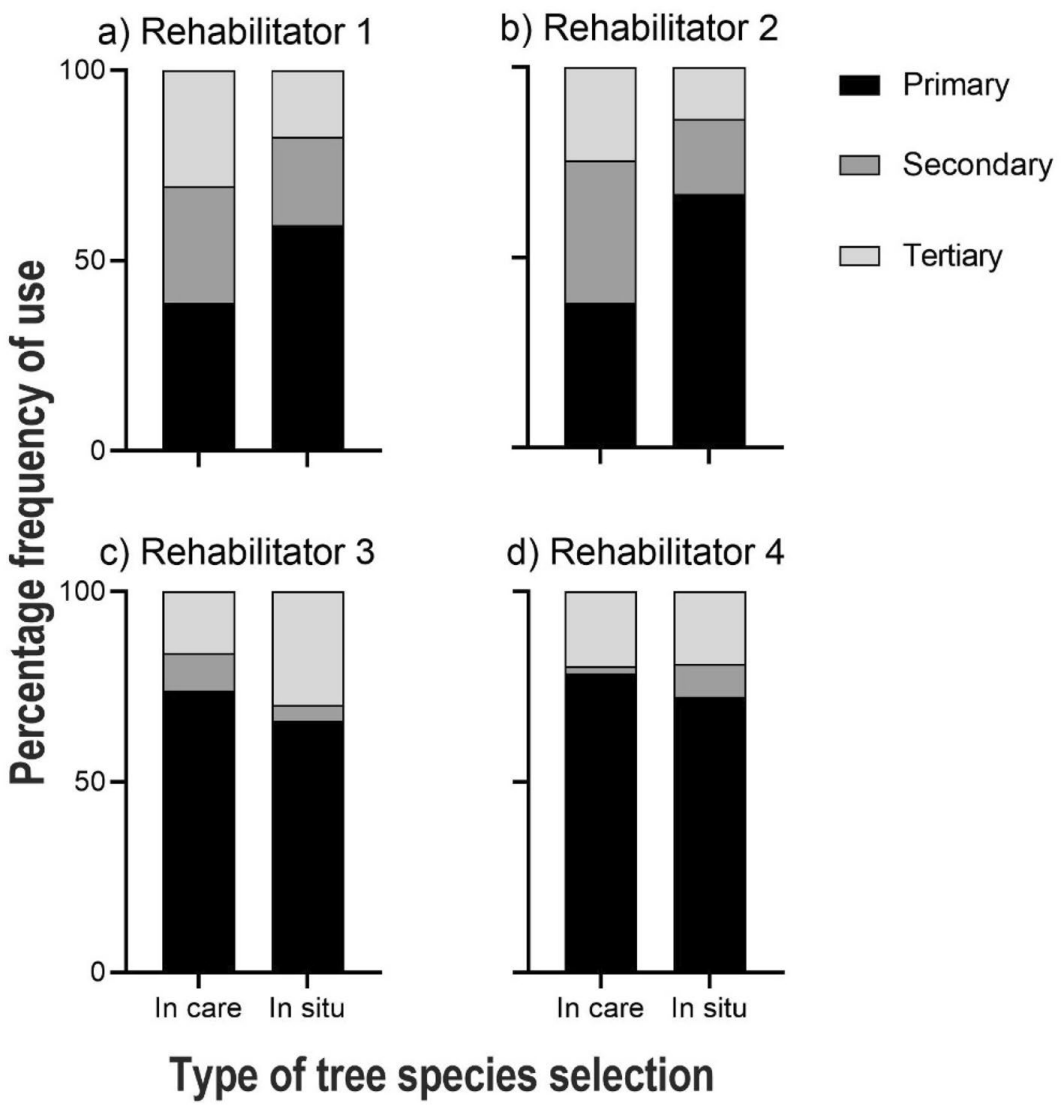
Comparison of the proportional distribution of tree species selected by rehabilitators for browse collection (n = 29 species) against the tree species selected by koalas in situ (n = 30 species). Tree species were grouped together based on importance to koalas, from primary to tertiary food trees, and all koalas for each rehabilitator were grouped.

We collected tree use data from 482 locations from 31 koalas monitored in situ after release from rehabilitation. Three koalas were in care for one night or less across all captures, and therefore weren’t considered, and we were not able to collect data on species fed in care for an additional two. We gathered floristic data from 98 browse collection sites used by the rehabilitators. Comparison of the relative frequency of food tree classes used for browse collection versus koala use in situ revealed a significant difference for all four koala rehabilitators (Rehabilitator 1, *X*^2^_2_ = 16.28, *p* < 0.001; Rehabilitator 2, *X*^2^_2_ = 12.48, *p* = 0.002; Rehabilitator 3, *X*^2^_2_ = 6.85, *p* = 0.030; Rehabilitator 4, *X*^2^_2_ = 11.78, *p* = 0.003; Fig. 6a–d respectively).

Across all rehabilitators and koalas a total of 43% (n = 40) of tree species were uniquely utilised; either used only by koalas in the wild but not fed in care (23%) or fed in care but not recorded as being used by koalas (20%). Importantly, Rehabilitators 1 and 2 fed browse from fewer primary food trees than koalas used in the wild (Fig. 6a,b). Rehabilitator 3 fed an approximately equivalent proportion of foliage from the primary food tree types, while their koalas used more tertiary-ranked tree species than other koalas (Fig. 6c). Rehabilitator 4 fed foliage from more primary and fewer secondary tree species (Fig. 6d). There was a significant positive linear relationship between the number of tracking locations for individual koalas and tree species richness (*R*^2^ = 0.642, *F*_(1,34)_ = 61.013, *p* < 0.001). Figure S1 shows the breakdown of trees species use by koalas in situ compared to browse fed in care.

**Figure 7:**
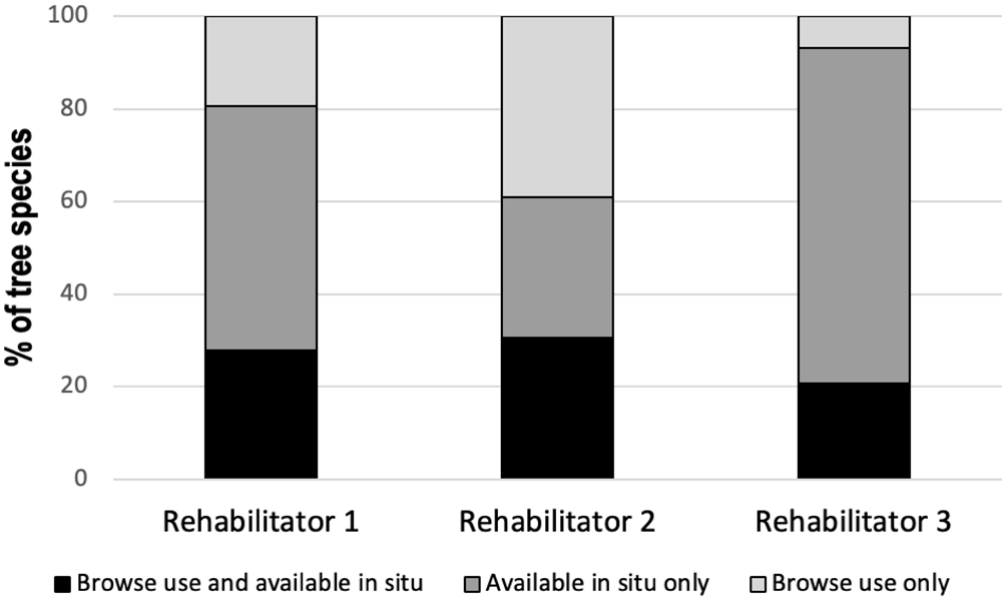
Comparison of the percentage of tree species used for browse collection that were also available to koalas in situ, versus those that were available to koalas in situ but not used for browse, and those that were used for browse but not available to koalas in situ.

Our comparison of the available tree species in the vegetation communities used by koalas after release, compared to the tree species used for browse collection by rehabilitators, revealed similar results. For all rehabilitators, 30% or less of species were both available to koalas in situ and also used for browse collection (Fig. 7).

## Discussion

Our study identified several critical areas where rehabilitation and release procedures can be improved, which are easily implemented and applicable to a range of different species. Our study found an annual survival rate of 58.5%, comparable to survival rates of other rehabilitated koalas (e.g., 58% survival^43^), but lower than some wild living populations (e.g., 92.5–74% survival^77^) which indicates that techniques for improving outcomes for koalas in care are much needed. We discuss a range of recommendations below, which contribute to the identified need for evidence-based and context specific protocols to maximise outcomes for rehabilitated animals^78^. We found that including basic assessments that don’t require any specialist equipment (e.g., body condition and climbing ability scores) can improve post-release survival rates. For koalas we recommend the inclusion of both measurements as a prerequisite before releasing animals from care.

Body condition indices are a commonly used method for determining the health and fitness of an animal^79^ and have sometimes been used to assess if animals have re-established post-release^80^, yet standardisation across practitioners can be challenging and may not always be considered a determining factor for suitability for release from care (e.g., the koala might be released if other indicators of health are positive). We note that there was a relatively small difference between body condition scores of koalas that survived the first 30 days (average 3.5 out of 5) and those that were recorded as mortalities (average 2.9), which would not always be consistent nor easy to differentiate. Further, although the Code of Practice requires that a rehabilitated koala’s pre-release body condition be no less than a 3 out of 5, four koalas were released with body condition scores that we assessed as 2.5, and two of these died (10 and 11 days after release). Body condition scoring of any species is subjective by nature but can be improved and standardised by providing professional development and training to volunteer rehabilitators and veterinarians along with regular evaluation of their skills^81^. Standardised protocols are also important for all species where this technique is applied. In addition, to improve outcomes, we recommend a conservative approach when determining if an animal with a body condition score on the cusp should be released, including consideration of other measurements (e.g., climbing ability), and regular evaluations to ensure compliance with assessment protocols.

Animals in captivity can lose condition and muscle tone due to long-term lack of mobility^82^, which in the case of koalas affects their climbing ability. Poor climbing ability directly affects koalas’ safety, as well as access to browse high in the canopy, and potentially their ability to move to preferred tree species. In this study, climbing ability was assessed as part of the study while the koala was being released and this score was found to be related to koala survival, and yet rarely would a koala be recaptured by rehabilitators if its climbing ability was poor. If systematic observations of an animal’s behaviour and physical condition can occur before release, they can be used to determine the animal’s fitness, and ultimately their preparedness for release. This can be achieved using either soft-release enclosures (an enclosed area in the animal’s natural habitat), or easy-access tree yards (in close proximity to rehabilitators for regular observation) with the additional benefits that the animals can re-acclimate to weather conditions and practice natural behaviours (e.g., climbing). Given the two week critical survival threshold we identified after release, the implementation of such simple precautions could have a substantial impact on reducing mortality rates. At the time of this study, while some rehabilitators had access to these enclosures, neither type was readily available to the majority of rehabilitators and were likely to be beyond the financial capacity of volunteers, necessitating support from the animal welfare sector or regulator.

Importantly, we found that the two weeks immediately following a koala’s release were critical to their survival. Monitoring is costly, particularly if the study subject is a cryptic arboreal animal^45^, and financial limitations are one of the main reasons for the lack of post-release monitoring studies^83^. The two-week critical survival threshold in our study suggests that, where resources are limited, there is high value in short-term, intensive, monitoring of rehabilitated animals to detect abnormal behaviours, injury, or poor condition, which can be indicators for intervention. Based on our results, monitoring animals in the immediate period following release is beneficial as it can inform protocols for pre-release assessments and determining release suitability. Additionally, our assessment of initial post-release movements suggest that there is an initial displacement response and these longer-distance movements carry increased risk for the animal, particularly in developed areas. Male koalas were more likely to die after care than females, which may be influenced by higher conspecific competition^84,85^ forcing greater movements and therefore higher energetic costs and risks in the urban landscape. Mortality was highest in the first year of the study, which included extreme weather events and heat stress around the time of the 2019–2020 Australian bushfires.

We identified that gaps in the level of information available to scientists and policy makers compared to wildlife rehabilitators has the potential to impact post-release success. For example, the Code of Practice recommends that koalas be released at their capture site, or as near as possible in a suitable environment, yet koalas were released, on average, 1395 m from their capture site and most koalas were released onto a different vegetation group than their capture site, with 39% released onto a lower quality habitat. Our results confirm the importance of this recommendation; koalas released further from their original capture site moved greater distances in the first 30 days (and further from their release site) following their release and, in an urban/peri-urban environment, increased movement may increase the probability of an adverse event to occur (e.g., vehicle collision or dog attack). Similar threats are likely to impact a range of species as the wildlife urban interface continues to grow across many countries^86,87^. However, we found a weak effect where the risk of mortality decreased when koalas were released further from their capture site, suggesting that rehabilitators were effective in assessing risk and changing release location accordingly. Non-compliance from volunteer wildlife rehabilitators on this recommendation is likely due to a range of factors including: a lack of access to guidance on alternative locations to release koalas when their original capture site was in a hazardous area; a lack of accessible data for people who may be unfamiliar with GIS map software typically used to identify suitable vegetation communities; and limited access to scientific literature on koala habitat quality at each site. We recommend that policy makers, scientists and wildlife rehabilitation organisations collaborate to review protocols and include decision making tools for dealing with complex release scenarios as part of wildlife rehabilitator training.

All mortalities occurred in koalas that received medical intervention (major and minor), with no mortalities for koalas that did not receive any medical intervention. This is perhaps unsurprising for the koalas that received major medical intervention, given these were koalas that were relatively seriously sick/injured. However, two out of the five koalas that died in the first two weeks of release only received minor medical intervention, with an additional ‘minor’ male koala being classed as a mortality in this time period as he subsequently required major medical intervention nine days after he was initially released (and eventually died). Two of these koalas had not been assessed by a veterinarian, only receiving oral fluids for rehydration from the rehabilitator and therefore may have had underlying conditions that were not detected. Easy access to veterinary expertise is an important consideration in this sector. We recommend that all koalas captured by volunteers are required to be assessed by a veterinarian, and we recognise this may require additional resources particularly since volunteer rehabilitators are caring for animals at their homes and the required expertise for thorough medical examination and treatment may be some distance away.

Interestingly, our results suggested that the duration of medical care and rehabilitation was not a significant issue in regard to mortality rates. The results indicated that the most important factors to consider pre-release were around judgements made on animal condition at the time of release, and there are relatively easy to apply assessments to aid in this decision-making stage and improve survival outcomes.

Koalas are dietary specialists and gut microbiome changes, due to changes in the diet, are thought to be critical limitations for translocation success rates^16,35,88^. A recent study using gut reinoculation of koalas in care suggested that changes in gut microbiota can drive a change in browse selection by koalas after release^89^ and such microbiota changes could potentially be driven by a change in diet in care^16^, particularly for koalas staying longer in care. However, in contrast, anecdotal evidence from wildlife rehabilitators suggests that koalas cope well with diet changes if they are moved from a poorer to a better-quality diet. An emergency evacuation of koalas from approaching bushfire in 2020 supported this idea, with the change in diet during a 3 month stay in captivity not adversely impacting their body condition or post-release survival over more than 12 months (unpublished data). Thus, this is a complex area that requires further study.

In this study, we found limited overlap between diets fed in care compared to the koala’s diets following their release. Due to the lack of documentation detailing which tree species were used to feed each koala, we assumed that all the tree species that were collected by wildlife rehabilitators were used to feed each koala regardless of duration in care. This assumption likely over-estimated the diversity of trees that were used to feed individual koalas. Similarly, we found a significant positive, linear relationship between the number of koala tracking locations and the number of tree species they were recorded using. Our results revealed a high number of tree species that koalas used that were not fed in care, including important food trees, and this data was based on limited, short-term monitoring of some koalas as many returned to care. If the duration of monitoring each koala was increased the number of species utilised by koalas in situ but not fed in care would be likely to increase, further broadening the gap between in situ and ex situ diets. To evaluate the impact of a difference in wild versus captive diet on the mortality or success of rehabilitated animals, the relationship between diet and mortality rates during care would ideally need to be considered. Since this study focused only on koalas that were successfully rehabilitated to release stage, that then have access to in situ tree species, that question was beyond scope but it is an area we recommend for further research given the potential impact of diet on koala health. An appropriate ex situ diet that meets an animal’s nutritional needs and is reflective of their wild diet is a well-established basis of animal husbandry^90^.

This captive versus wild dietary gap is important not only for rehabilitated animals of various species, but for other conservation scenarios including headstarting and translocations which are increasingly being considered a management tool for endangered species^28^, as habitats become more fragmented by development and climate change induces further population declines. Recent studies of other species indicate that questions around the impact of diet and other variables on the microbiome of animals in captivity are varied and complex^91–94^ and this is an important area for further study.

There is a paucity of information on how rehabilitation practices impact animals’ post-release survival and re-establishment in the wild, likely reflecting a historical divide between the scientific community and the volunteer wildlife rehabilitation sector^83^. Given the large effort and resources required to rehabilitate animals, we recommend rehabilitation practices be regularly examined, across different species, to ensure effective allocation of resources. This study provides insights into improving outcomes for rehabilitated koalas and, while most relevant for arboreal folivores, provides a model, including the types of determining factors to consider, for other post-rehabilitation monitoring programs.

## Supplementary Information

Supplementary Information.

## Data Availability

The datasets analysed and the code developed during the current study are available from the corresponding author on request.
