# Supplementary material for: Outcomes for an arboreal folivore after rehabilitation and implications for management
Source: Sci Rep. 2023 Apr 21;13:6542. doi: 10.1038/s41598-023-33535-y (PMC10121558; doi:10.1038/s41598-023-33535-y)
Supplement: Supplementary file 1 — Supplementary Information. [file 41598_2023_33535_MOESM1_ESM.docx]

# Supplementary material

Outcomes for an arboreal folivore after rehabilitation and implications for management.

Kellie A. Leigh, Lacey N. Hofweber, Brienna K. Sloggett, Victoria L. Inman, Lachlan J Pettit, Aditi Sriram, Ron Haering

**Text S1**

Throughout this report, publicly available vegetation maps were used to determine the vegetation community utilised by each koala post-release (accessed via https://datasets.seed.nsw.gov.au). The vegetation maps overlapped in areas, so they were preferentially ranked for use based on accuracy of ground-truthing carried out under this project. The maps were ranked in the following descending order:

1. Remnant vegetation of the western Cumberland subregion, 2013 Update. VIS_ID 4207 (OEH, 2013)
2. The native vegetation of the Sydney metropolitan area - Version 3. VIS_ID 4489 (OEH, 2016)
3. Hawkesbury City Council vegetation mapping, 2007. VIS_ID 3958 (OEH, 2007)
4. Draft vegetation map, south eastern Wollemi National Park, 2010. VIS_ID 4184 (OEH, 2010)
5. Native vegetation mapping in the Blue Mountains 1999-2002. VIS_ID 2239 (DECCW, 2011)

In instances where multiple vegetation types were assigned to a single koala location waypoint, the higher-ranking vegetation map was selected.

DECCW. (2011). *Native vegetation mapping in the Blue Mountains 1999-2002 VIS_ID 2239*. NSW Department of Environment,Climate Change and Water.

OEH. (2007). *Hawkesbury City Council Vegetation Mapping, 2007. VIS_ID 3958*. Office of Environment and Heritage.

OEH. (2010). *Draft Vegetation map, South Eastern Wollemi National Park, 2010. VIS_ID 4184*. Office of Environment and Heritage.

OEH. (2013). *Remnant Vegetation of the western Cumberland subregion, 2013 Update. VIS_ID 4207*. Office of Environment and Heritage.

OEH. (2016). *The Native Vegetation of the Sydney Metropolitan Area - Version 3 VIS_ID 4489*. Office of Environment and Heritage.

*Table S1. Vegetation groupings and the vegetation types that comprise them. Maps accessed via* [*https://datasets.seed.nsw.gov.au*](https://datasets.seed.nsw.gov.au)

| Vegetation Group | Vegetation type | Vegetation Map |
| --- | --- | --- |
| Alluvial Woodland | Alluvial Woodland | CumberlandPlainWest_2013_E_4207.shp |
|  | Cumberland Riverflat Forest | SydneyMetroArea_v3_1_2016_E_4489 |
| Hawkesbury Sandstone Gully Forest | Sydney Sandstone Gully Forest | HawkesburyLGA_2007_E_3958 |
|  | Sydney Sandstone Gully Forest-Open Forest | HawkesburyLGA_2007_E_3958 |
| Sandstone Gully Forest | Coastal Sandstone Gully Forest | SydneyMetroArea_v3_1_2016_E_4489 |
|  | Sydney Sandstone Gully Forest | Baulkham Hills Map |
| Shale Sandstone Transition Forest | Shale Sandstone Transition Forest (Low Sandstone Influence) | CumberlandPlainWest_2013_E_4207.shp |
|  | Shale Sandstone Transition Forest (High Sandstone Influence) | CumberlandPlainWest_2013_E_4207.shp |
|  | Shale Sandstone Transition Forest (Low Sandstone Influence) | HawkesburyLGA_2007_E_3958 |
|  | Shale Sandstone Transition Forest (High Sandstone Influence) | HawkesburyLGA_2007_E_3958 |
|  | Cumberland Shale-Sandstone Ironbark Forest | SydneyMetroArea_v3_1_2016_E_4489 |
| Shale Woodlands | Shale Plains Woodland | CumberlandPlainWest_2013_E_4207.shp |
|  | Moist Shale Woodland | CumberlandPlainWest_2013_E_4207.shp |
|  | Shale Hills Woodland | CumberlandPlainWest_2013_E_4207.shp |
|  | Cumberland Shale Hills Woodland | SydneyMetroArea_v3_1_2016_E_4489 |
|  | Cumberland Shale Plains Woodland | SydneyMetroArea_v3_1_2016_E_4489 |
| Sydney Hinterland Apple Forest | Sydney Hinterland Peppermint-Apple Forest | WollemiNP_SthEastDraft_E_4184 |
|  | Sydney Hinterland Sheltered Turpentine-Apple Forest | WollemiNP_SthEastDraft_E_4184 |
|  | Sydney Hinterland Sheltered Turpentine-Apple Forest | HawkesburyNorthernDraft08_E_4167 |
|  | Sydney Hinterland Peppermint Apple Forest | HawkesburyNorthernDraft08_E_4167 |
| Sydney Hinterland Bloodwoods | Sydney Hinterland Bloodwood Mahagony Forest | HawkesburyNorthernDraft08_E_4167 |
|  | Sydney Hinterland Rocky Yellow Bloodwood Woodland | HawkesburyNorthernDraft08_E_4167 |
| Sydney Sandstone Ridgetop Woodland | Sandstone Ridgetop Woodland | CumberlandPlainWest_2013_E_4207.shp |
|  | Sydney Sandstone Ridgetop Woodland | HawkesburyLGA_2007_E_3958 |
|  | Sydney Sandstone Ridgetop Woodland-Open Forest | HawkesburyLGA_2007_E_3958 |
|  | Sydney Sandstone Ridgetop Woodland-Open Woodland | HawkesburyLGA_2007_E_3958 |
|  | Sydney Hinterland Dwarf Apple Scrub | HawkesburyNorthernDraft08_E_4167 |
|  | Exposed Scribbly Gum Forest | HawkesburyNorthernDraft08_E_4167 |
| Turpentine Ironbark | Turpentine-Ironbark Margin Forest | CumberlandPlainWest_2013_E_4207.shp |
|  | Turpentine-Ironbark Forest | CumberlandPlainWest_2013_E_4207.shp |
|  | Turpentine-Ironbark Forest | HawkesburyLGA_2007_E_3958 |
|  | Turpentine-Ironbark Margin Forest | HawkesburyLGA_2007_E_3958 |
|  | Sydney Turpentine-Ironbark Forest | SydneyMetroArea_v3_1_2016_E_4489 |
| Upper Georges River Sandstone Woodland | Upper Georges River Sandstone Woodland | CumberlandPlainWest_2013_E_4207.shp |
|  | Upper Georges River Sandstone Woodland | HawkesburyLGA_2007_E_3958 |
| Western Sandstone Gully Forest | Western Sandstone Gully Forest | CumberlandPlainWest_2013_E_4207.shp |
|  | Sydney Hinterland Apple-Blackbutt Gully Forest | SydneyMetroArea_v3_1_2016_E_4489 |
| Not grouped | *Corymbia gummifera - Corymbia eximia* | Blue_mountains_LGA_VISmap_2239 |
|  | *Corymbia gummifera - Eucalyptus sparsifolia* | Blue_mountains_LGA_VISmap_2239 |
|  | Riparian Woodland | CumberlandPlainWest_2013_E_4207.shp |
|  | Riparian Forest | CumberlandPlainWest_2013_E_4207.shp |
|  | Western Sydney Dry Rainforest | CumberlandPlainWest_2013_E_4207.shp |
|  | Floodplain Woodland (Roberts 1999) | HawkesburyLGA_2007_E_3958 |
|  | Transition Woodland (Roberts 1999) | HawkesburyLGA_2007_E_3958 |
|  | Sydney South Exposed Sandstone Woodland | SydneyMetroArea_v3_1_2016_E_4489 |
|  | Sydney Ironstone Bloodwood-Silvertop Ash Forest | SydneyMetroArea_v3_1_2016_E_4489 |
|  | Sydney Hinterland Exposed Sandstone Woodland | SydneyMetroArea_v3_1_2016_E_4489 |
|  | Sydney Hinterland Grey Gum Ridgetop Forest | SydneyMetroArea_v3_1_2016_E_4489 |
|  | Sydney Hinterland Dwarf Apple Heath-Woodland | SydneyMetroArea_v3_1_2016_E_4489 |
|  | Coastal Enriched Sandstone Moist Forest | SydneyMetroArea_v3_1_2016_E_4489 |
|  | Coastal Shale-Sandstone Forest | SydneyMetroArea_v3_1_2016_E_4489 |
|  | Blue Mountains Blue Gum-Turpentine Gully Forest | WollemiNP_SthEastDraft_E_4184 |
|  | Blue Mountains Grey Gum-Stringybark Transition Forest | WollemiNP_SthEastDraft_E_4184 |
|  | Lower Blue Mountains Exposed Red Bloodwood Woodland | WollemiNP_SthEastDraft_E_4184 |
|  | Sydney Hinterland Warm Temperate Rainforest | WollemiNP_SthEastDraft_E_4184 |

**Text S2**

model1 <- glmmTMB(DistDaily ~

poly(DaysSinceRelease,2) +

Vegdif +

Soildif +

KHSMdif +

LogDistanceBetweenCaptureReleases +

LogDaysInCare +

Age +

Sex +

MedicalIntervention +

poly(DaysSinceRelease,2):Vegdif +

poly(DaysSinceRelease,2):Soildif +

poly(DaysSinceRelease,2):KHSMdif +

poly(DaysSinceRelease,2):LogDistanceBetweenCaptureReleases +

poly(DaysSinceRelease,2):LogDaysInCare +

poly(DaysSinceRelease,2):Age +

poly(DaysSinceRelease,2):Sex +

poly(DaysSinceRelease,2):MedicalIntervention +

poly(DaysSinceRelease,2):LogDistanceBetweenCaptureReleases:LogDaysInCare +

poly(DaysSinceRelease,2):LogDistanceBetweenCaptureReleases:Age +

poly(DaysSinceRelease,2):LogDistanceBetweenCaptureReleases:Sex +

poly(DaysSinceRelease,2):LogDistanceBetweenCaptureReleases:MedicalIntervention +

poly(DaysSinceRelease,2):LogDaysInCare:Age +

poly(DaysSinceRelease,2):LogDaysInCare:Sex +

poly(DaysSinceRelease,2):LogDaysInCare:MedicalIntervention +

poly(DaysSinceRelease,2):Age:Sex +

poly(DaysSinceRelease,2):Age:MedicalIntervention +

poly(DaysSinceRelease,2):Sex:MedicalIntervention +

(1|KoalaName), family = Gamma(link = "log"), Data)

*Table S2. Classification of Eucalyptus, Angophora and Corymbia species used by koalas into primary, secondary, and tertiary food trees.*

*Trees were classified based on level of importance to koalas as listed in:*

*NSW Office of Environment and Heritage. A review of koala tree use across New South Wales. Office of Environment and Heritage, 2018, and*

*Phillips, S and J. Callahan (2000) Tree species preferences of koalas (Phascolarctos cinereus) in the Campbelltown area south-west of Sydney, New South Wales. Wildlife Research 27, 509-516

Since koalas are known to use different tree species in different areas, we added and ranked tree species based on a review of two local datasets; one for Campbelltown and South West Sydney (n>50 adult koalas) ^76^ (Close, R. Campbelltown koala research and database.* [*https://campbelltownkoalaresearchanddatabase.com*](https://campbelltownkoalaresearchanddatabase.com)*, 2019) and unpublished data for the Wollemi and Hawkesbury area (n>30 adult koalas). Tree species were classified as primary if they comprised 25% or more of records by >15% of koalas at either study site, or were used by more than 25% of koalas that had at least 10 location records. Secondary tree species were used by more than 17% of koalas and Supplementary tree species comprised more than 10% of records for more than one koala. Both areas contained high vegetation diversity and over 20 tree species used by koalas at each site (from genus Eucalyptus, Angophora and Corymbia).*

| **Primary** | **Secondary** | **Tertiary** | |  |
| --- | --- | --- | --- | --- |
| *A. costata* | *A. floribunda* | | *E. haemastoma* | |
| *C. gummifera* | *A. bakeri* | | *C. eximia* | |
| *E. agglomerata* | *E. crebra* | | *C. maculata* | |
| *E. amplifolia* | *E. deanei* | | *E. botryioides* | |
| *E. pilularis* | *E. fibrosa* | | *E. camaldulensis* | |
| *E. piperita* | *E. moluccana* | | *E. capitellata* | |
| *E. punctata* | *E. nicholli* | | *E. cinerea* | |
| *E. tereticornis* | *E. sclerophylla* | | *E. citriodora* | |
|  | *E. sieberi* | | *E. eugenioides* | |
|  | *E. viminalis* | | *E. floribunda* | |
|  |  | | *E. globoidea* | |
|  |  | | *E. leucoxylon* | |
|  |  | | *E. longifolia* | |
|  |  | | *E. microcorys* | |
|  |  | | *E. notabilis* | |
|  |  | | *E. oblonga* | |
|  |  | | *E. parramattensis* | |
|  |  | | *E. resinifera* | |
|  |  | | *E. robusta* | |
|  |  | | *E. saligna* | |
|  |  | | *E. sclerophylla* | |
|  |  | | *E. scorparia* | |
|  |  | | *E. sideroxlyn* | |
|  |  | | *E. sparsifolia* | |
|  |  | | *S. glomulifera* | |

*Table S3. Number of koalas in each age and sex category by medical intervention.*

| Medical intervention | Adult | | Subadult | |
| --- | --- | --- | --- | --- |
|  | M | F | M | F |
| None | 1 | 1 | 2 | 2 |
| Minor | 9 | 4 | 1 | 2 |
| Major | 7 | 3 | 2 | 2 |

*Table S4. Top ranked models examining drivers of daily distance moved by koalas in the first 30 days following release. All models were family Gamma (log = link) and included koala ID as a random variable.*

| **Daily distance moved in first 30 days ~** | **df** | **logLik** | **AICc** | **ΔAICc** |
| --- | --- | --- | --- | --- |
| poly(DaysSinceRelease, 2) + LogDistanceBetweenCaptureReleases | 6 | -776.6 | 1565.9 | 0.00 |
| poly(DaysSinceRelease, 2) + LogDistanceBetweenCaptureReleases + Age | 7 | -775.7 | 1566.2 | 0.26 |
| poly(DaysSinceRelease, 2) + LogDistanceBetweenCaptureReleases + Sex | 7 | -775.9 | 1566.7 | 0.78 |
| poly(DaysSinceRelease, 2) + LogDistanceBetweenCaptureReleases + SoilDiff | 7 | -776.3 | 1567.5 | 1.64 |
| poly(DaysSinceRelease, 2) + LogDistanceBetweenCaptureReleases*Age | 9 | -774.1 | 1567.7 | 1.77 |
| poly(DaysSinceRelease, 2) + LogDistanceBetweenCaptureReleases + VegDiff | 7 | -776.5 | 1567.8 | 1.86 |
| poly(DaysSinceRelease, 2) + LogDistanceBetweenCaptureReleases + Age + Sex | 8 | -775.4 | 1567.8 | 1.92 |

*Table S5. Top ranked models examining drivers of the maximum distance koalas were from their release site in the first 30 days following release.*

| **Maximum distance from release site in first 30 days ~** | **df** | **logLik** | **AICc** | **ΔAICc** |
| --- | --- | --- | --- | --- |
| LogDistanceBetweenCaptureReleases | 3 | -31.9 | 71.0 | 0.00 |
| LogDistanceBetweenCaptureReleases + MedicalIntervention | 5 | -29.4 | 72.0 | 0.98 |
| LogDistanceBetweenCaptureReleases + Sex | 4 | -31.4 | 72.8 | 1.74 |

*Table S6. Top ranked models examining drivers of the maximum distance koalas were from their release site in the first 100 days following release.*

| **Maximum distance from release site in first 100 days ~** | **df** | **logLik** | **AICc** | **ΔAICc** |
| --- | --- | --- | --- | --- |
| LogDistanceBetweenCaptureReleases + Sex | 4 | -18.2 | 48.1 | 0.00 |
| 1 | 2 | -21.7 | 48.4 | 0.27 |
| Sex | 3 | -20.7 | 49.5 | 1.36 |
| LogDistanceBetweenCaptureReleases | 3 | -20.8 | 49.6 | 1.45 |
| MedicalIntervention | 3 | -20.8 | 49.6 | 1.51 |

Figure S1. Percentage occurrence that each Eucalyptus, Angophora, and Corymbia tree species was fed to koalas in care compared to what koalas used in situ, for each rehabilitator.


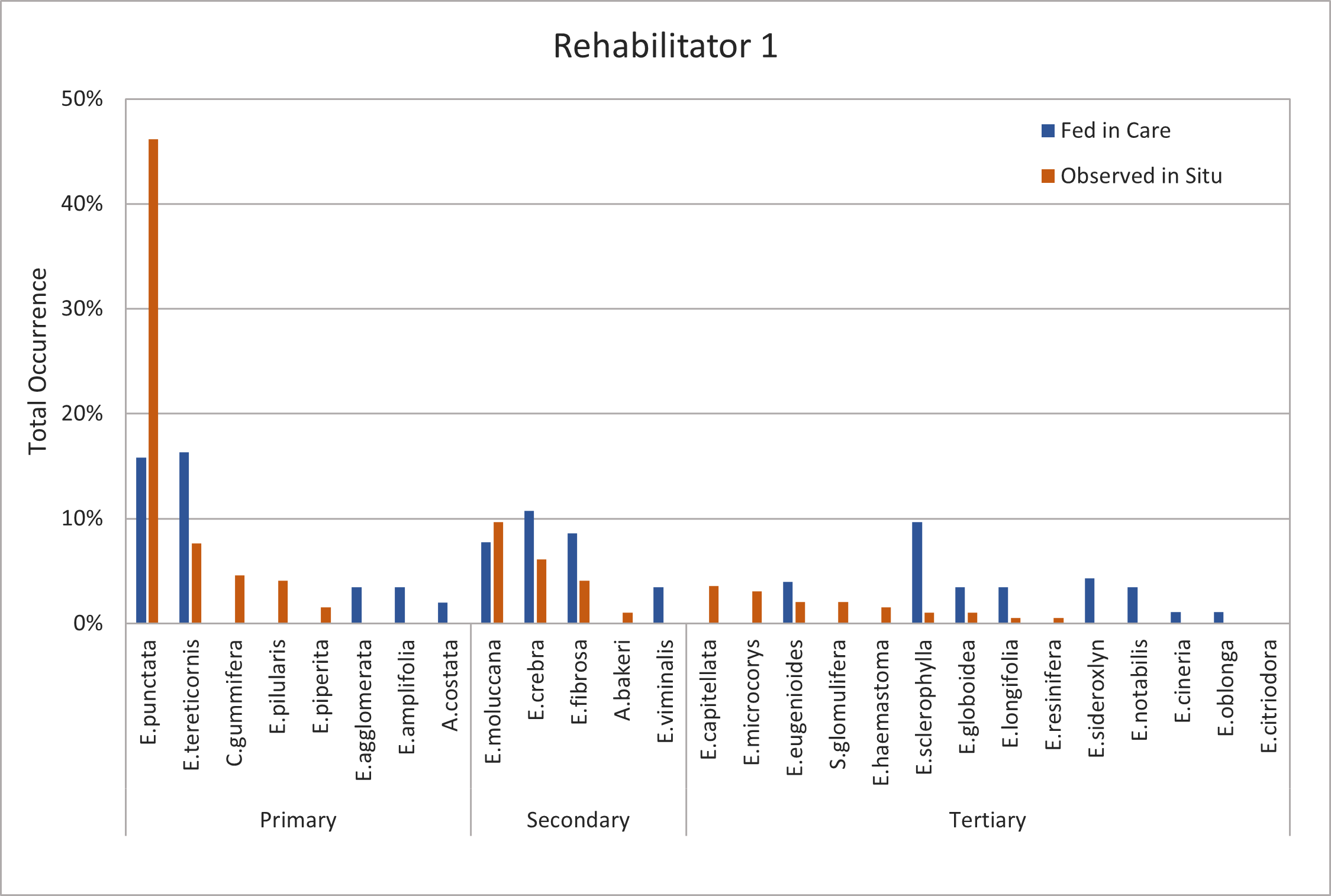


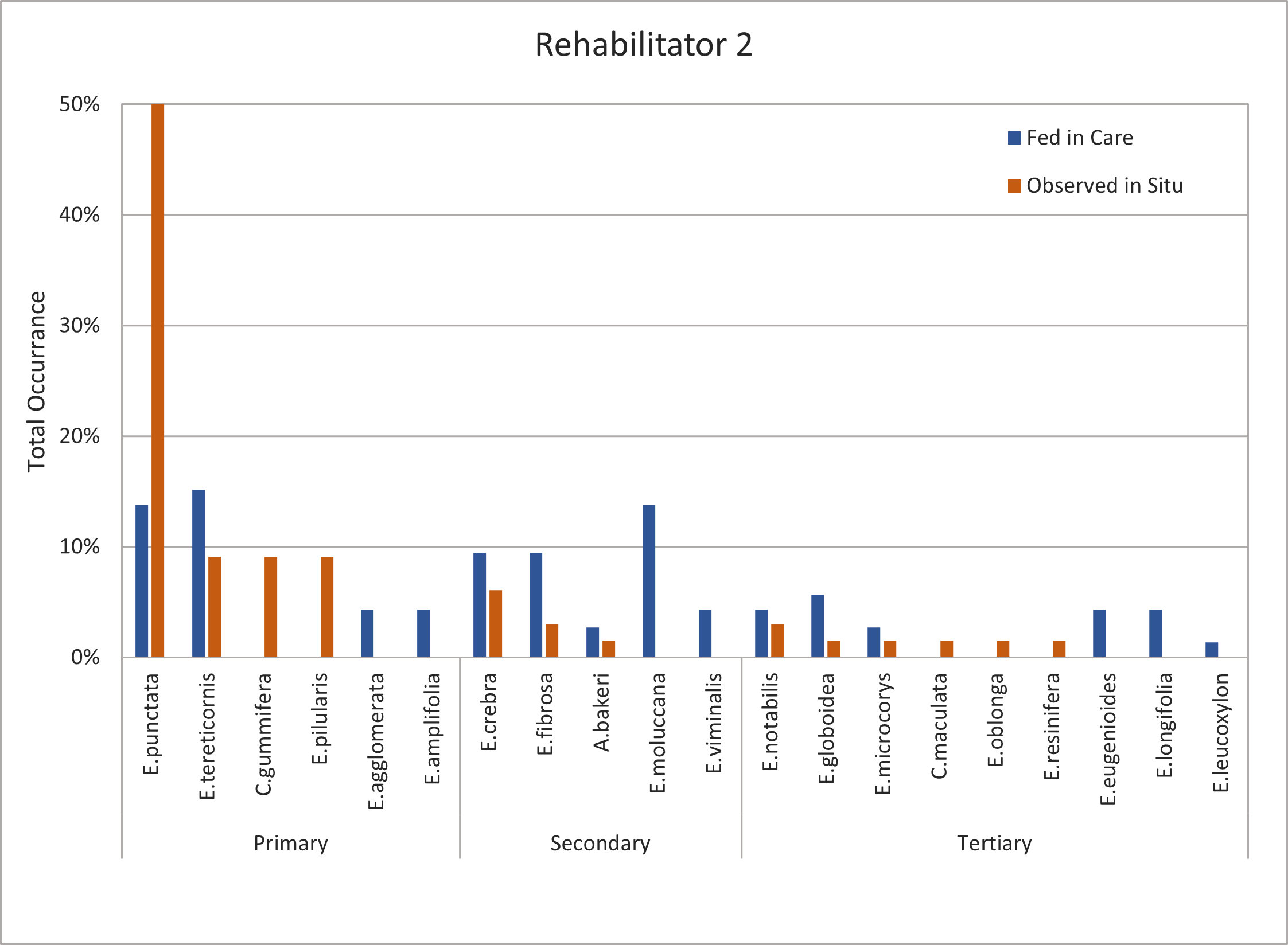


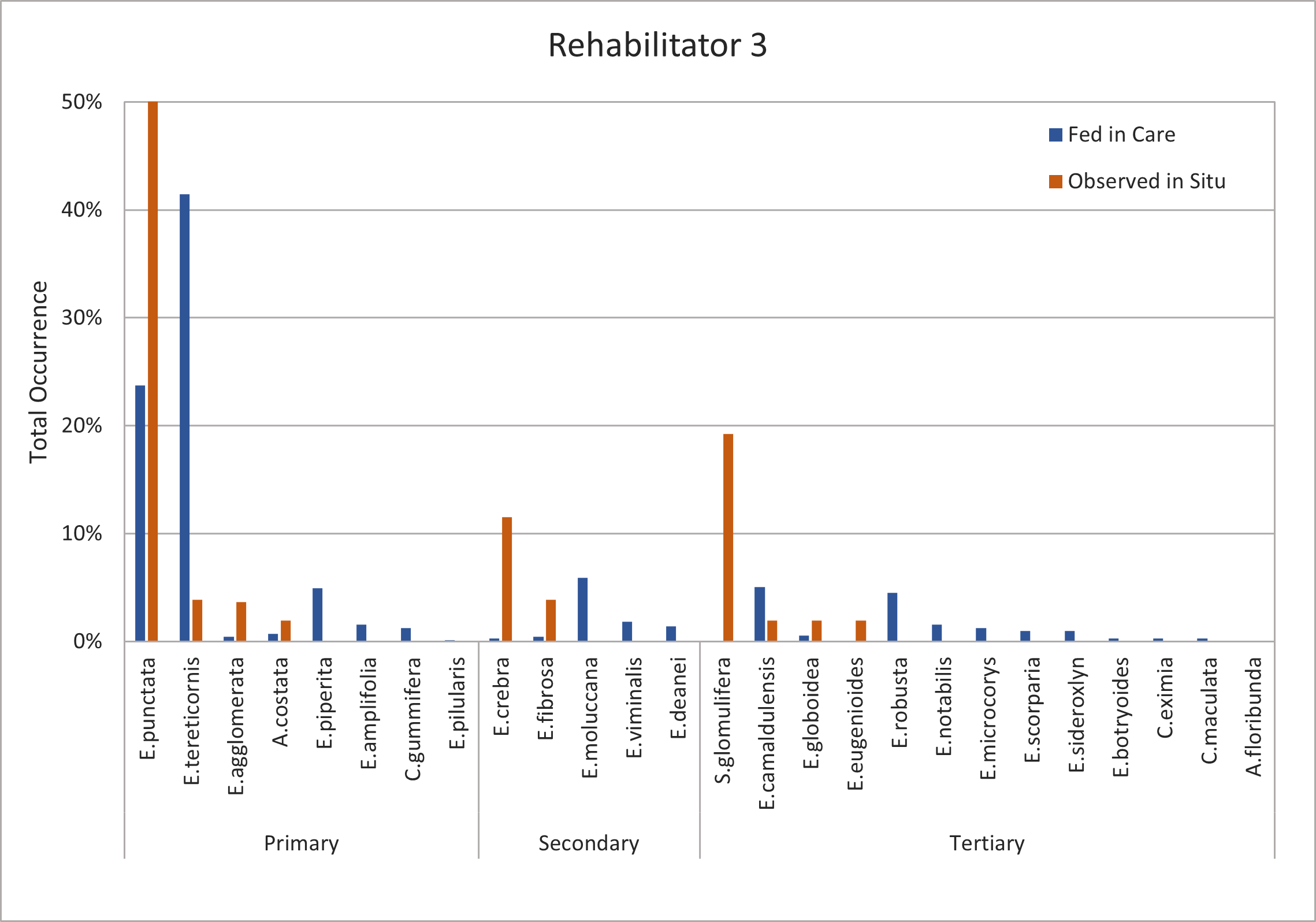


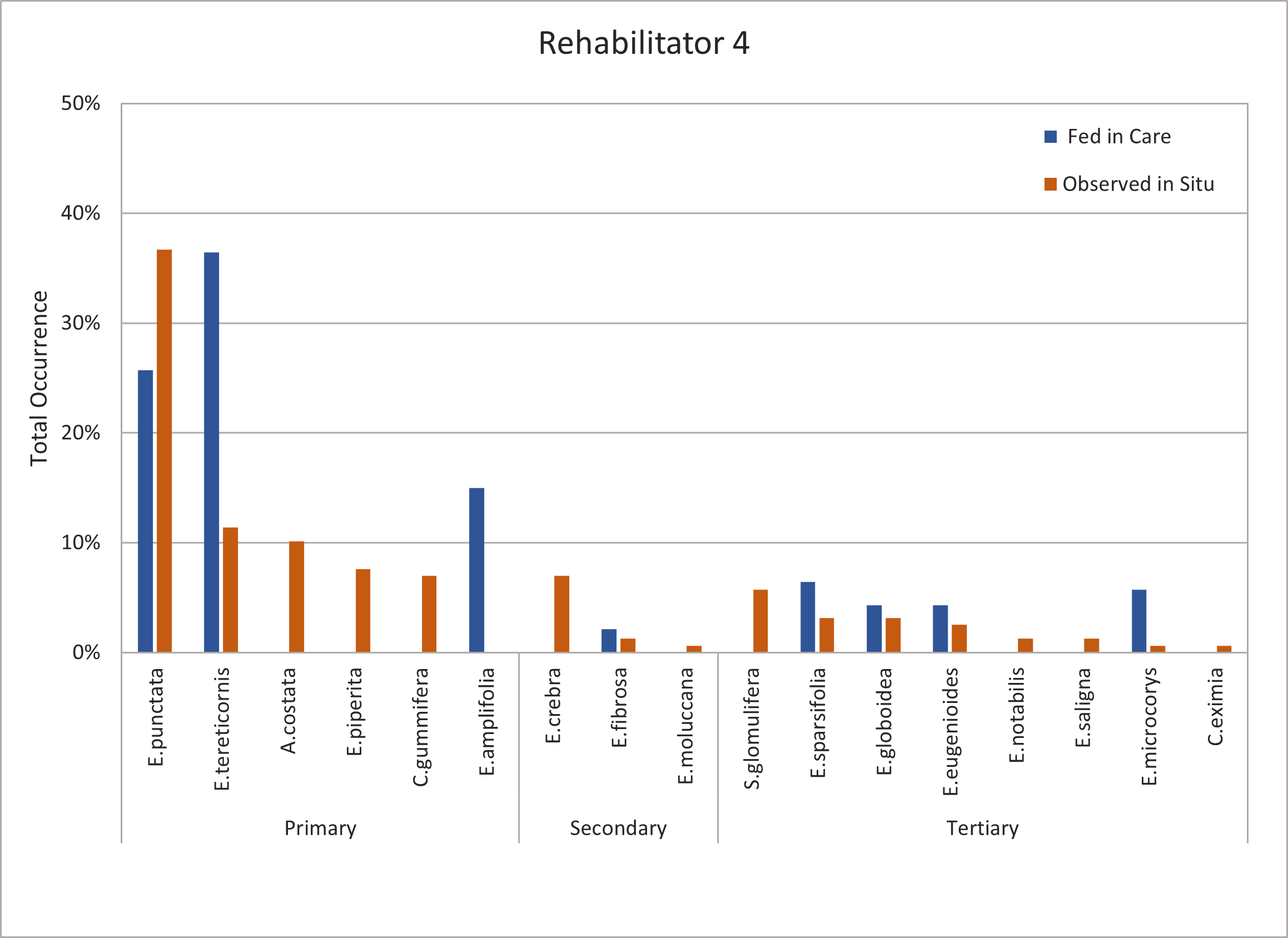


Table S7. Details of koala history and fate

| **Koala** | **Capture** | **Year of study** | **Region** | **Age group** | **Sex** | **Date captured** | **Reason for capture** | **Medical intervention** | **Details of medical treatment** | **Days in care** | **Date Released** | **Last date tracked** | **Days in situ** | **Distance between capture and release sites (m)** | **Body condition score (at time of release)** | **Climbing ability score (at time of release) 1-3** | **Survival** | **Survival analysis days** | **Survival analysis medical intervention** | **Comments** | **Capture history prior to this study** |
| --- | --- | --- | --- | --- | --- | --- | --- | --- | --- | --- | --- | --- | --- | --- | --- | --- | --- | --- | --- | --- | --- |
| Aimee | First | 2 | SW | Adult | F | 28/9/2021 | Imminent danger (private property with dogs) | None | None. Not taken to vet. | 1 | 29/9/2021 | 1/11/2021 | 33 | 568 | 3.5 | 3 | Survived | 33 | None |  | N |
| Annie | First | 2 | NW | Adult | F | 3/10/2020 | Suspected chlamydia | Major | Sedated for health assessment and swabbed for chlamydia (positive), treated with antibiotics. | 64 | 6/12/2020 | 22/6/2021 | 198 | 85 | 3 | 3 | Survived | 198 | Major |  | N |
| Aries | First | 2 | SW | Adult | F | 26/4/2021 | Sitting on ground, irritated eye | Minor | Oral fluids and criticare for dehydration. Sedated and swabbed for chlamydia (negative). | 12 | 8/5/2021 | 30/7/2021 | 83 | 175 | 3 | 2.5 | Survived | 161 | Minor |  | N |
|  | Second |  |  |  |  | 30/7/2021 | Imminent danger (roadside) | Minor | Sedated for health assessment and swabbed for chlamydia (negative). Relaxed pouch muscle and joey undernourished, so monitored in care. Joey eventually died. | 56 | 24/9/2021 | 11/12/2021 | 78 | 256 | 3 | Not assessed |  |  |  |  |  |
| Bobby | First | 1 | SW | Adult | M | 7/1/2020 | Imminent danger (private property with dogs) | Minor | Not taken to vet. Oral fluids for dehydration. Monitored for infection due to a possible dog bite. | 5 | 12/1/2020 | 21/1/2020 | 9 | 274 | 3 | 1 | Mortality | 9 | Minor | Treated as mortality (returned to care for Major medical treatment) on 21/01/2020. Later, he was re-released (12/02/2020), re-rescued (13/02/2020), and then died in care (24/02/2020). Cause of death: bone marrow myeloid and erythroid hypoplasia, marked lymphoid depletion (spleen) | N |
|  | Second |  |  |  |  | 21/1/2020 | Sitting on ground | Major | Sedated for health assessment and swabbed for chlamydia (negative). Bacterial infection treated with antibiotics. IV fluids for dehydration. | 22 | 12/2/2020 | 13/2/2020 | 1 | 606 | 3 | 2 |  |  |  |  |  |
|  | Third |  |  |  |  | 13/2/2020 | Sitting on ground | Major | Sedated for health assessment. Bacterial infection treated with antibiotics. |  | Died in care | Not applicable | Not applicable | Not applicable | Not applicable | Not applicable |  |  |  |  |  |
| Boris | First | 2 | SW | Subadult | M | 26/11/2020 | Suspected dog attack | Major | Superficial injuries to eye, nose and mouth. Sedated for health assessment and swabbed for chlamydia (negative). Injections of anti-inflammatory and antiobiotics. | 20 | 16/12/2020 | 23/11/2021 | 342 | 731 | 3.5 | 3 | Survived | 360 | Major |  | N |
|  | Second |  |  |  |  | 23/11/2021 | Imminent danger (roadside) | None | None. Not taken to vet. | 0 | 23/11/2021 | 11/12/2021 | 18 | 822 | Not assessed | Not assessed |  |  |  |  |  |
| Cammi | First | 1 | SW | Adult | M | 15/1/2020 | Imminent danger (roadside), heat stress | Minor | Sedated for health assessment and swabbed for chlamydia (negative). Oral fluids for dehydration. Biopsy on lumps on groin (benign). | 10 | 25/1/2020 | 2/2/2020 | 8 | 589 | 3 | 2 | Mortality | 8 | Minor | Mortality in care on 07/02/2020. Mortality date recorded as date put back into care (02/02/2020). Cause of death: died coming out of anaesthesia. | N |
|  | Second |  |  |  |  | 2/2/2020 | Sitting on ground, heat stress, severe dehydration, irritated eye | Major | IV fluids for dehydration. Surgery to remove benign lumps in groin. |  | Died in care | Not applicable | Not applicable | Not applicable | Not applicable | Not applicable |  |  |  |  |  |
| Daisy | First | 2 | SW | Subadult | F | 13/8/2021 | Imminent danger (roadside), laceration near eye | Minor | Sedated for health assessment and swabbed for chlamydia (negative). Stitches to laceration. | 8 | 21/8/2021 | 11/12/2021 | 112 | 1130 | 4 | 3 | Survived | 112 | Minor |  | N |
| Digger | First | 2 | SW | Subadult | M | 29/9/2021 | Imminent danger (roadside) | None | None. Not taken to vet. | 3 | 2/10/2021 | 12/10/2021 | 10 | 751 | 3 | 3 | Survived | 59 | None |  | N |
|  | Second |  |  |  |  | 12/10/2021 | Imminent danger (roadside) | None | None. Not taken to vet. | 0 | 12/10/2021 | 24/10/2021 | 12 | 281 | Not assessed | Not assessed |  |  |  |  |  |
|  | Third |  |  |  |  | 24/10/2021 | Imminent danger (roadside) | None | None. Not taken to vet. | 1 | 25/10/2021 | 1/12/2021 | 37 | 1210 | Not assessed | Not assessed |  |  |  |  |  |
| Ella | First | 2 | SW | Adult | F | 5/7/2021 | Imminent danger (roadside) | Minor | Sedated for health assessment and swabbed for chlamydia (negative). | 4 | 9/7/2021 | 23/7/2021 | 14 | 1045 | 3 | 2 | Mortality | 28 | Minor | Eventually deemed unsuitable for release in wild, placed in permanent care at Port Macquarie Koala Hospital. Was calssified as a mortality on 6/08/2021 due to requiring major medical intervention | Y |
|  | Second |  |  |  |  | 23/7/2021 | Imminent danger (roadside) | None | None. Not taken to vet. | 0 | 23/7/2021 | 6/8/2021 | 14 | 2597 | 3.5 | Not assessed |  |  |  |  |  |
|  | Third |  |  |  |  | 6/8/2021 | Imminent danger (roadside) | Major | Sedated for health assessment. Swollen toe, one claw missing. Injections of anti-inflammatory drug. | 13 | 19/8/2021 | 28/8/2021 | 9 | 1315 | Not assessed | Not assessed |  |  |  |  |  |
|  | Fourth |  |  |  |  | 28/8/2021 | Unsuitable environment (private property) | None | None. Not taken to vet. Monitored for climbing ability. | 20 | 17/9/2021 | 19/9/2021 | 2 | 4884 | Not assessed | Not assessed |  |  |  |  |  |
|  | Fifth |  |  |  |  | 19/9/2021 | Imminent danger (roadside) | None | None. Not taken to vet. Monitored for climbing ability. | 44 | 2/11/2021 | 5/12/2021 | 33 | 3412 | Not assessed | Not assessed |  |  |  |  |  |
|  | Sixth |  |  |  |  | 5/12/2021 | In same tree for 12 days, imminent danger (roadside) |  |  |  | Not released | Not applicable | Not applicable | Not applicable | Not applicable | Not applicable |  |  |  |  |  |
| Ernie | First | 1 | NW | Adult | M | 22/2/2020 | Suspected chlamydia | Major | Sedated for health assessment and swabbed for chlamydia (positive), treated with antibiotics. One eye removed. Topical antifungals for hair loss on feet due to suspected fungal dermatitis. | 154 | 25/7/2020 | 8/11/2020 | 106 | 222 | 2.5 | 3 | Survived | 106 | Major | Had injuries related to collar so was recaptured and survival date calculated as rescue date (8/11/2020). Returned to wild without tracking device. Not considered a mortality for survival analysis | N |
|  | Second |  |  |  |  | 8/11/2020 | Complication with tracking device | Minor | Sedated for health assessment and swabbed for chlamydia (negative). Wound on neck, treated with topical antibacterial. | 6 | 14/11/2020 | Not applicable | Not applicable | Not applicable | Not assessed | Not assessed |  |  |  |  |  |
| Fernando | First | 1 | NW | Adult | M | 13/4/2020 | Suspected chlamydia | Major | Sedated for health assessment and swabbed for chlamydia (positive), treated with antibiotics. | 83 | 5/7/2020 | 23/12/2020 | 171 | 1019 | 3.5 | 3 | Survived | 171 | Major | Had injuries related to collar so was recaptured and survival date calculated as rescue date (23/12/2020). Not considered a mortality for survival analysis. Died 25/12/2020. | N |
|  | Second |  |  |  |  | 23/12/2020 | Complication with tracking device | Major | Large flesh wound on neck, treated with antibiotics. Maggots removal from wound and washed with iodine. |  | Died in care | Not applicable | Not applicable | Not applicable | Not applicable | Not applicable |  |  |  |  |  |
| Gilly | First | 1 | NW | Adult | F | 27/9/2020 | Suspected chlamydia | Major | Sedated for health assessment and swabbed for chlamydia (positive), treated with antibiotics. | 70 | 6/12/2020 | 31/3/2021 | 115 | 1822 | 2.5 | 3 | Survived | 115 | Major |  | Y |
| Gladys | First | 1 | SW | Adult | F | 29/1/2020 | Imminent danger (roadside) | Minor | Sedated for health assessment and swabbed for chlamydia (negative). While in care developed eye infection, treated with antibacterial cream. | 23 | 21/2/2020 | 1/6/2020 | 101 | 3210 | 4 | 3 | Survived | 169 | Minor | For survival analysis, Gladys was censored on the 3rd capture date (08/08/2020) as she went into care for a long time due to twins, but did not herself receive life saving medical intervention. | Y |
|  | Second |  |  |  |  | 1/6/2020 | Imminent danger (roadside) | None | None. Not taken to vet. | 0 | 1/6/2020 | 8/8/2020 | 68 | 815 | 4.5 | 3 |  |  |  |  |  |
|  | Third |  |  |  |  | 8/8/2020 | Had twins, both underweight | Minor | Sedated for health assessment. Treated for mastitis. | 378 | 21/8/2021 | 26/12/2021 | 127 | 66 | 3.5 | 3 |  |  |  |  |  |
| Gwen | First | 2 | NW | Adult | F | 9/4/2021 | Suspected chlamydia | Major | Sedated for health assessment and swabbed for chlamydia (positive), treated with antibiotics. Unilateral paraovarian cyst detected and drained. Ear discharge treated with antibiotic injection. | 125 | 12/8/2021 | 28/10/2021 | 77 | 128 | 3 | 2 | Survived | 77 | Major |  | Y |
| Herman | First | 2 | NW | Adult | M | 27/2/2021 | Suspected chlamydia | Major | Sedated for health assessment and swabbed for chlamydia (positive), treated with antibiotics. | 57 | 25/4/2021 | 8/9/2021 | 136 | 208 | 3 | 2 | Mortality | 136 | Major | Treated as mortality (returned to care for Major medical treatment) on 08/09/2021 | N |
|  | Second |  |  |  |  | 8/9/2021 | Suspected chlamydia | Major | Sedated for health assessment and swabbed for chlamydia (positive), treated with antibiotics. | 45 | 23/10/2021 | 14/12/2021 | 52 | 216 | Not assessed | Not assessed |  |  |  |  |  |
| Huntress | First | 2 | NW | Subadult | F | 14/1/2020 | Rescued during bushfire | Major | Sedated for health assessment and swabbed for chlamydia (negative). Maggots in cloaca flushed out. Bacterial infection treated with antibiotics. Oral and IV fluids for dehydration. | 129 | 22/5/2020 | 14/12/2021 | 571 | 4020 | 3.5 | 3 | Survived | 571 | Major |  | N |
| Jarod | First | 1 | NW | Subadult | M | 28/12/2019 | Burns to paws, dehydration | Major | Sedated for health assessment and swabbed for chlamydia (negative). Burns treated with topical antibacterial cream. IV fluids for dehydration. | 102 | 8/4/2020 | 11/4/2020 | 3 | 370 | 3.5 | 1 | Mortality | 3 | Major | Mortality in situ on 11/04/2020. Cause of death: presumed predation - all internal organs had been eaten so wasn’t possible to assess underlying disease | N |
| Jeff | First | 1 | SW | Adult | M | 1/2/2020 | Sitting on ground, severely heat stressed | Major | Sedated for health assessment and swabbed for chlamydia (negative). Oral and IV fluids for dehydration. | 123 | 3/6/2020 | 13/6/2020 | 10 | 858 | 2.5 | 3 | Mortality | 10 | Major | Mortality in care on 18/06/2020. Mortality date recorded as date put back into care (13/06/2020). Cause of death: died coming out of anaesthesia. | N |
|  | Second |  |  |  |  | 13/6/2020 | Imminent danger (roadside), wheezing, irritated eye |  | Sedated for health assessment. Mass found in lung. |  | Died in care | Not applicable | Not applicable | Not applicable | Not applicable | Not applicable |  |  |  |  |  |
| Julie | First | 1 | SW | Subadult | F | 16/12/2020 | Imminent danger (roadside) | None | None. Not taken to vet. | 1 | 17/12/2020 | 3/1/2021 | 17 | 730 | 4 | 3 | Survived | 17 | None |  | Y |
| Kai | First | 1 | SW | Adult | M | 21/11/2020 | Imminent danger (roadside) | None | None. Not taken to vet. | 0 | 21/11/2020 | 30/11/2020 | 9 | 620 | 2.5 | 2 | Mortality | 11 | Minor | Mortality in situ on 04/12/2020. Cause of death: head and muscle trauma, generalised cachexia | N |
|  | Second |  |  |  |  | 30/11/2020 | Sitting on ground | Minor | Not taken to vet. Oral fluids for dehydration. | 2 | 2/12/2020 | 4/12/2020 | 2 | 46 | Not assessed | Not assessed |  |  |  |  |  |
| Kellie | First | 2 | NW | Subadult | F | 22/12/2019 | Rescued during bushfire | Major | Sedated for health assessment and swabbed for chlamydia (negative). Topical creams for skin condition. IV fluids for dehydration. Pneumonia treated with antibiotics. | 152 | 22/5/2020 | 30/11/2021 | 557 | 306 | 4 | 3 | Survived | 557 | Major |  | N |
| Kevin | First | 1 | SW | Adult | M | 13/12/2020 | Unsuitable environment (private property) | Minor | Not taken to vets. Oral fluids for rehydration. | 2 | 15/12/2020 | 29/5/2021 | 165 | 301 | 3 | 3 | Survived | 167 | Major | Not considered a mortality as even though he received major medical treatment in subsequent release, we do not consider this to be life saving medical intervention | N |
|  | Second |  |  |  |  | 29/5/2021 | Unsuitable environment (private property), blood on left eye | Major | Sedated for health assessment. Grass seed deep in eye removed and treated with antibiotics. | 13 | 11/6/2021 | 13/6/2021 | 2 | 354 | 4.5 | 3 |  |  |  |  |  |
| Kobi | First | 2 | SW | Adult | M | 2/8/2021 | Dehydration, poor condition | Major | Sedated for health assessment and swabbed for chlamydia (negative). IV fluids for dehydration. | 9 | 11/8/2021 | 11/12/2021 | 122 | 776 | 3.5 | 3 | Survived | 122 | Major |  | N |
| Merlin | First | 2 | SW | Adult | M | 5/3/2021 | Unsuitable environment (far from known koala population), trouble climbing | Major | Sedated for health assessment. IV fluids for dehydration. Diagnosed with hip dysplasia. Stomach bloating treated with cisapride. | 148 | 31/7/2021 | 29/8/2021 | 29 | 9104 | 4 | 2 | Survived | 29 | Major |  | N |
| Missy | First | 2 | SW | Subadult | F | 10/9/2021 | Imminent danger (roadside) | Minor | Not taken to vets. Oral fluids for dehydration. | 7 | 17/9/2021 | 9/12/2021 | 83 | 2120 | 3.5 | 3 | Survived | 85 | Minor |  | N |
|  | Second |  |  |  |  | 9/12/2021 | Imminent danger (roadside) | None | None. Not taken to vet. | 0 | 9/12/2021 | 11/12/2021 | 2 | 1984 | 3.5 | 3 |  |  |  |  | N |
| Modi | First | 2 | SW | Adult | F | 13/4/2021 | Sitting on ground | Minor | Sedated for health assessment and swabbed for chlamydia (negative). Unknown mass in stomach that cleared. | 25 | 8/5/2021 | 11/12/2021 | 217 | 1846 | 3 | 3 | Survived | 217 | Minor |  | Y |
| Moe | First | 2 | SW | Subadult | M | 15/9/2021 | Imminent danger (roadside) | None | None. Not taken to vet. | 0 | 15/9/2021 | 11/12/2021 | 87 | 947 | 4 | 3 | Survived | 87 | None |  | N |
| Peaches | First | 2 | SW | Subadult | F | 5/11/2021 | Imminent danger (private property with dogs) | None | None. Not taken to vet. Monitoring for signs of dog attack. | 6 | 11/11/2021 | 11/12/2021 | 30 | 129 | 3.5 | 2 | Survived | 30 | None |  | N |
| Roger | First | 2 | NW | Adult | M | 15/8/2020 | Suspected chlamydia | Major | Sedated for health assessment and swabbed for chlamydia (positive), treated with antibiotics. | 98 | 21/11/2020 | 19/3/2021 | 118 | 23 | 3 | 3 | Mortality | 118 | Major | Mortality in situ on 19/03/2021. Cause of death: undetermined, suspected diagnosis Lymphoma (renal) | N |
| Royal | First | 1 | SW | Adult | M | 21/6/2020 | Imminent danger (roadside) | Minor | Sedated for health assessment and swabbed for chlamydia (negative). Cut foot in care, treated with topical antibacterial cream. | 46 | 6/8/2020 | 23/11/2020 | 109 | 1328 | 3.5 | 1 | Survived | 109 | Minor |  | N |
| Ruben | First | 2 | SW | Subadult | M | 5/10/2019 | Imminent danger (roadside) | Minor | Sedated for health assessment and swabbed for chlamydia (negative). Oral fluids for rehydration. Bilateral cataracts, corrected themselves during care. | 378 | 17/10/2020 | 13/6/2021 | 239 | 4616 | 4 | 3 | Survived | 239 | Minor |  | N |
| Sam | First | 2 | SW | Adult | M | 7/6/2021 | Imminent danger (roadside) | Minor | Sedated for health assessment and swabbed for chlamydia (negative). | 4 | 11/6/2021 | 25/6/2021 | 14 | 856 | 4.5 | 3 | Survived | 181 | Minor |  | N |
|  | Second |  |  |  |  | 25/6/2021 | Imminent danger (roadside) | None | None. Not taken to vet. | 0 | 25/6/2021 | 8/7/2021 | 13 | 1749 | 4 | 3 |  |  |  |  |  |
|  | Third |  |  |  |  | 8/7/2021 | Imminent danger (roadside) | None | None. Not taken to vet. | 1 | 9/7/2021 | 22/7/2021 | 13 | 2834 | Not assessed | Not assessed |  |  |  |  |  |
|  | Fourth |  |  |  |  | 22/7/2021 | Imminent danger (roadside) | None | None. Not taken to vet. | 1 | 23/7/2021 | 18/8/2021 | 26 | 3939 | 4.5 | Not assessed |  |  |  |  |  |
|  | Fifth |  |  |  |  | 18/8/2021 | Imminent danger (roadside) | None | None. Not taken to vet. | 0 | 18/8/2021 | 11/12/2021 | 115 | 192 | Not assessed | Not assessed |  |  |  |  |  |
| Simmo | First | 2 | SW | Adult | M | 8/9/2021 | Apparent shoulder injury, eye infection | Minor | Sedated for health assessment. Eye infection treated with topical cream. | 16 | 24/9/2021 | 5/12/2021 | 72 | 3696 | 3 | 2.5 | Survived | 72 | Minor |  | N |
| Wally | First | 1 | NW | Adult | M | 26/6/2020 | Imminent danger (roadside) | Minor | Sedated for health assessment and swabbed for chlamydia (negative). | 15 | 11/7/2020 | 27/12/2020 | 169 | 3878 | 4.5 | 3 | Survived | 464 | Minor |  | Y |
|  | Second |  |  |  |  | 27/12/2020 | Unsuitable environment (private property) | None | None. Not taken to vet. | 0 | 27/12/2020 | 18/10/2021 | 295 | 2217 | 3.5 | Not assessed |  |  |  |  |  |
| Waminda | First | 1 | SW | Adult | M | 3/9/2020 | Imminent danger (roadside) | Minor | Sedated for health assessment and swabbed for chlamydia (negative). | 16 | 19/9/2020 | 2/6/2021 | 256 | 499 | 4 | 1 | Mortality | 256 | Minor | Mortality in care. Mortality date is when he was put back into care, he died on 19/07/2021. Note that between 29/09/2020 and 2/06/2021 Waminda was not tracked | N |
|  | Second |  |  |  |  | 2/6/2021 | Plant spikes in paws | Major | Sedated for health assessment. Wounds on paws cleaned with topical antibacterial cream. Anti-inflammatory injections and antibiotics administered. |  | Died in care | Not applicable | Not applicable | Not applicable | Not applicable | Not applicable |  |  |  |  |  |
| Will | First | 2 | SW | Adult | M | 24/5/2020 | Imminent danger (private property with dogs), blood on nose | Minor | Sedated for health assessment and swabbed for chlamydia (negative). | 10 | 3/6/2020 | 22/10/2020 | 141 | 1237 | 3.5 | 2 | Survived | 141 | Minor | Note that between 24/06/2020 and 22/10/2021 Will was not tracked | N |
